# Consistency of the full and reduced order models for evolve‐filter‐relax regularization of convection‐dominated, marginally‐resolved flows

**DOI:** 10.1002/nme.6942

**Published:** 2022-03-25

**Authors:** Maria Strazzullo, Michele Girfoglio, Francesco Ballarin, Traian Iliescu, Gianluigi Rozza

**Affiliations:** ^1^ mathLab, Mathematics Area SISSA Trieste Italy; ^2^ Department of Mathematics and Physics Catholic University of the Sacred Heart Brescia Italy; ^3^ Department of Mathematics Virginia Tech Blacksburg Virginia USA

**Keywords:** evolve‐filter‐relax stabilization, marginally‐resolved convection‐dominated flows, Navier–Stokes equations, proper orthogonal decomposition, reduced order modeling

## Abstract

Numerical stabilization is often used to eliminate (alleviate) the spurious oscillations generally produced by full order models (FOMs) in under‐resolved or marginally‐resolved simulations of convection‐dominated flows. In this article, we investigate the role of numerical stabilization in reduced order models (ROMs) of marginally‐resolved, convection‐dominated incompressible flows. Specifically, we investigate the FOM–ROM consistency, that is, whether the numerical stabilization is beneficial both at the FOM and the ROM level. As a numerical stabilization strategy, we focus on the evolve‐filter‐relax (EFR) regularization algorithm, which centers around spatial filtering. To investigate the FOM‐ROM consistency, we consider two ROM strategies: (i) the EFR‐noEFR, in which the EFR stabilization is used at the FOM level, but not at the ROM level; and (ii) the EFR‐EFR, in which the EFR stabilization is used both at the FOM and at the ROM level. We compare the EFR‐noEFR with the EFR‐EFR in the numerical simulation of a 2D incompressible flow past a circular cylinder in the convection‐dominated, marginally‐resolved regime. We also perform model reduction with respect to both time and Reynolds number. Our numerical investigation shows that the EFR‐EFR is more accurate than the EFR‐noEFR, which suggests that FOM‐ROM consistency is beneficial in convection‐dominated, marginally‐resolved flows.

## 1 INTRODUCTION

In the numerical simulation of incompressible fluid flows modeled by the Navier–Stokes equations (NSE), standard full order models (FOMs) (e.g., finite element (FE) methods, finite volume (FV) methods, finite difference (FD) methods or spectral elements (SE)) work well in the *resolved* regime, that is, when the number of degrees of freedom is large enough to represent the complex flow dynamics. However, FOMs are generally not accurate in the *under‐resolved* regime, that is, when the number of degrees of freedom is too low to represent the flow dynamics. We note that FOMs are also inaccurate in the *marginally‐resolved* regime, when the number of degrees of freedom is just enough to capture the flow dynamics. However, the FOM inaccuracy is less pronounced in the marginally‐resolved regime than in the under‐resolved regime. A classic illustration of under‐resolved and marginally‐resolved FOMs is in the *convection‐dominated* regime (i.e., at high Reynolds numbers, when the viscosity is low and the convective term dominates the diffusion term), when the FOM mesh‐size is larger than the smallest flow scales. In this case, the FOMs generally yield *spurious numerical oscillations* that significantly degrade the FOM numerical accuracy.

To eliminate or alleviate the spurious numerical oscillations of the FOMs in convection‐dominated, under‐resolved and marginally‐resolved simulations, various *numerical stabilization* methods have been proposed over the years. Many of these FOM numerical stabilization approaches are surveyed in the research monograph of Roos et al..^1^ *Regularized* models are a popular class of numerical stabilization methods in which *spatial filtering* is used to regularize (smooth) various terms in the NSE and eliminate (alleviate) the spurious numerical oscillations of the FOMs in convection‐dominated, under‐resolved and marginally‐resolved simulations. A plethora of regularized models are surveyed in the research monograph of Layton and Rebholz.^2^ A classical regularized model is the *Leray* model (proposed in 1934 by the great mathematician Jean Leray^3^), which regularizes the convective field in the NSE nonlinearity. Another classical regularized model is the *evolve‐filter‐relax (EFR)* model, which consists of three steps: (i) in the “evolve” step, a standard FOM is used to obtain an intermediate approximation of the velocity; (ii) in the “filter” step, a spatial filter is used to filter (regularize) the intermediate approximation obtained in step (i) and eliminate (alleviate) its spurious numerical oscillations;^4^, ^5^, ^6^, ^7^, ^8^, ^9^, ^10^, ^11^, ^12^, ^13^ (iii) in the “relax” step, a more accurate velocity approximation is obtained as a convex combination between the filtered and unfiltered flow approximations.^14^, ^15^ The EFR model is a popular regularized model that has been used for different classical numerical methods, for example, the FE method^14^, ^16^ and the SE method.^7^ The main reasons for the popularity of the EFR model are its *simplicity* and *modularity*: given a legacy FOM code, the “evolve” step is already implemented, the “filter” step requires the addition of a simple subroutine, and the “relax” step is just one line of code.

To summarize the above discussion, when FOMs are used in the convection‐dominated, under‐resolved (marginally‐resolved) regime, regularized models (e.g., the Leray or the EFR models) can be used to eliminate (alleviate) the spurious numerical oscillations. In general, the need for numerical stabilization in the convection‐dominated, under‐resolved (marginally‐resolved) regime is well known and well documented in the realm of classical numerical methods: there are hundreds (if not thousands) of papers and several research monographs on this topic, and commercial software often includes numerical stabilization strategies for the under‐resolved (marginally‐resolved) regime. Our goal is to investigate this topic in a reduced order modeling context.

*Reduced order models (ROMs)* are relatively low‐dimensional computational models that can reduce the FOM computational cost by orders of magnitude.^17^, ^18^, ^19^, ^20^, ^21^, ^22^ The basic ROM idea is to collect solutions of the system computed for several parameter values, build a relatively low‐dimensional manifold, and then perform efficient simulations on this manifold for new parameter values. ROM strategies have been successfully applied in several contexts, from elliptic coercive problems^19^, ^22^ to Stokes flows^23^, ^24^ to nonlinear frameworks.^25^, ^26^, ^27^ The ultimate ROM goal is to make an impact in important applications (e.g., uncertainty quantification, shape optimization, flow control, and data assimilation), where numerical simulations need to be repeated for a large number of physical and/or geometrical parameter values. In these applications, ROMs could represent an efficient alternative to FOMs, whose computational cost is generally prohibitively high. We emphasize, however, that many of these practical applications take place in the convection‐dominated regime. Since in the convection‐dominated, under‐resolved (marginally‐resolved) regime numerical stabilization plays a central role for FOMs, a natural question is whether numerical stabilization is also beneficial in the ROM setting. To address this issue, one could first ask the following question:

**(Q1)** *Assuming that the FOM is run in the convection‐dominated, resolved regime and the ROM is run in the convection‐dominated, under‐resolved (marginally‐resolved) regime, is numerical stabilization needed for the ROM?*

For Galerkin projection based ROMs, it was shown in References 25, 28, 29, 30, 31, 32, 33 that, in the convection‐dominated regime, under‐resolved (marginally‐resolved) ROMs (i.e., ROMs in which the number of ROM basis functions is too low to capture all the flow scales) yield numerical oscillations even though the snapshots used to construct the ROMs were generated by FOMs used in a resolved regime (i.e., with a sufficiently large number of degrees of freedom to capture all the flow scales). Furthermore, it was also shown that *regularized ROMs (Reg‐ROMs)*, for example, the Leray ROM^33^, ^34^, ^35^ and the EFR‐ROM,^33^ can alleviate the spurious numerical oscillations and significantly increase the standard ROM accuracy. Finally, in Reference 33, it was shown that the EFR‐ROM was more accurate than the Leray ROM in the numerical simulation of a three‐dimensional flow past a cylinder. These results suggest that the answer to question (Q1) is that numerical stabilization is needed for Galerkin projection based ROMs in the convection‐dominated, under‐resolved (marginally‐resolved) regime and that Reg‐ROMs can alleviate the numerical oscillations and increase the accuracy of standard ROMs. (See Reference 36 for an alternative approach, based on a least‐squares Petrov–Galerkin projection.)

A natural follow‐up question to (Q1) is the following:

**(Q2)** *Assuming that both the FOM and the ROM are run in convection‐dominated, marginally‐resolved regime, if numerical stabilization is used for the FOM, is numerical stabilization still needed for the ROM?*

To our knowledge, question (Q2) is still open. In this article, we take a step in answering question (Q2). Specifically, we consider two scenarios in which both the FOM and the ROM are run in the marginally‐resolved regime. In this setting, we compare two types of ROMs:

(i) EFR‐noEFR, in which we use the EFR regularization at the FOM level but not at the ROM level; and
(ii) EFR‐EFR, in which we use the EFR regularization both at the FOM level and at the ROM level. In this article, we call this strategy *“FOM‐ROM consistency”*.

We compare the EFR‐noEFR and the EFR‐EFR in the numerical simulation of a 2D incompressible flow past a circular cylinder with time‐dependent Reynolds number.^37^, ^38^ The numerical results show that the EFR‐EFR is significantly more accurate than the EFR‐noEFR. Thus, these results suggest that the answer to question (Q2) is that, in the convection‐dominated, marginally‐resolved regime, numerical stabilization should be used both at a FOM level and at a ROM level, that is, that FOM‐ROM consistency is beneficial.

To our knowledge, this is the first time the FOM‐ROM consistency is investigated for the EFR regularization. The FOM‐ROM consistency has been advocated only in a few other settings, for example, for classical residual based stabilization methods^28^, ^30^, ^39^ and for a variational multiscale method.^40^ The FOM‐ROM consistency has also been investigated for a hybrid approach in Reference 41, where which the Leray model was combined with the EF algorithm. We emphasize that our study is different from the numerical investigation in Reference 41 in several key aspects.

1. The main difference between the two investigations is the algorithm used: we use the EFR algorithm, whereas in Reference 41 the authors use a combination of Leray and EF algorithms. In particular, in our current investigation we use the “relax” step, whereas the investigation in Reference 41 does not. This is a critical difference between the two investigations, since the “relax” step has been shown to be essential in increasing the algorithm's accuracy.^14^, ^15^
2. An important difference between the two investigations is that in the current study we perform the model reduction both in time and in the parametric space. Specifically, we leverage a nested proper orthogonal decomposition (POD) approach to develop ROMs that include variations with respect to the Reynolds number, which is a critical parameter in practical ROM applications. In contrast, the investigation in Reference 41 does not consider parametric variations with respect to the Reynolds number (although it includes a standard POD reduction strategy with parametric filter radius).
3. Another significant difference between the two investigations is the spatial discretization at a FOM level: in the current study we employ the FE method, whereas the investigation in Reference 41 uses the FV method. The FE and FV methods are two of the most used spatial discretizations in the numerical simulations of fluid flows. Since the FE and FV methods yield different ROM formulations (e.g., different ROM operators^42^), it is important to investigate the FOM‐ROM consistency in both settings.

Remark 1 Although enforcing FOM‐ROM consistency may seem a natural choice,^28^, ^43^, ^44^, ^45^ there exist numerous investigations that are FOM‐ROM inconsistent. For example, for turbulent flow simulations, there exist investigations that use a closure model at the FOM level, but not at the ROM level: On page 722 in Reference 46, the authors note that the FOM data is generated by using the AERO‐F code, which employs a DES turbulence model based on the Spalart–Allmaras one‐equation model. Instead of using a closure model, the ROM uses a least‐squares Petrov–Galerkin (LSPG) formulation. On page 17 in Reference 47, the authors mention that the FOM data is generated by using Vreman's LES model. However, the ROM utilizes the LSPG formulation instead of a closure model. On page 15 in Reference 32 (see also Appendix A), the authors note that the FOM data is generated by using a DNS. However, at the ROM level, the authors use several closure models of LES type (e.g., the dynamic SGS model). In Reference 48, the author employs a regularized model to generate the FOM data, and a data‐driven LES closure model to construct the ROM. On page 604 in Reference 33, the authors mention that the FOM data is generated by the same type of DNS as that used in Reference 32. However, at the ROM level, the authors employ two types of regularized ROMs, that is, the EF‐ROM and the Leray‐ROM. On page 317 in Reference 49, the authors note that RANS equations are used to generate the FOM data. However, the RANS equations are not used at the ROM level. There are, of course, examples of FOM‐ROM inconsistencies with respect to discretization choices other than closure, for example, time discretization,^32^, ^46^, ^50^ nonlinearity discretization,^51^ and stabilization. Examples of FOM‐ROM inconsistency with respect to stabilization include,^30^, ^39^ where the authors employ a FOM equipped with residual‐based stabilization, and discuss advantages (e.g., accuracy) and disadvantages (e.g., computational cost) of including such stabilization in the ROM. The FOM–ROM inconsistency with respect to stabilization is also investigated in the case of a residual‐based variational multiscale (VMS) formulation in Reference 40, where it is shown that dropping the VMS terms in the ROM formulation leads to a considerable deterioration of the ROM accuracy.

The reason for the relative popularity of the FOM–ROM inconsistency is most probably its practical convenience: to build the ROM, one is not restricted by the particular choices made in the FOM numerical discretization. Thus, the FOM–ROM inconsistency belongs to the general class of approaches that consider the FOM data and the ROM as different entities. In this article, we espouse a different line of thought in which the FOM and ROM are not completely independent. Specifically, we show numerically that building ROMs that are consistent with the FOMs with respect to the particular numerical regularization used can yield more accurate solutions.

The rest of the article is outlined as follows: In Section 2, we describe the FOM and the EFR algorithm. In Section 3, we focus on ROMs for time reduction, and compare the EFR‐noEFR and EFR‐EFR in the numerical simulation of a 2D flow past a circular cylinder. In Section 4, we compare the EFR‐noEFR and EFR‐EFR when model reduction is performed both in time and in the Reynolds number. Finally, in Section 5, we present conclusions and future research directions.

## 2 THE FULL ORDER MODEL AND THE EVOLVE‐FILTER‐RELAX ALGORITHM

In this section, we present the FOM and the EFR algorithm. As a mathematical model, we use the incompressible Navier–Stokes equations (NSE). Given a fixed domain $\Omega \subset {\mathbb{R}}^{D}$, with D=2,3, we consider the motion of an incompressible fluid having velocity u≐u(x,t)∈𝕌 and pressure p≐p(x,t)∈ℚ represented by the NSE:

(1) $$\left\{\begin{array}{cc} \frac{\partial u}{\partial t}+(u\cdot \nabla )u-\nu \Delta u+\nabla p=0\; & \text{in}\;\Omega \times (t_{0},T),\\ \nabla \cdot u=0\; & \text{in}\;\Omega \times (t_{0},T),\\ u=u_{D}\; & \text{on}\;\partial \Omega_{D}\times (t_{0},T),\\ -\;p\;n+\nu \frac{\partial u}{\partial n}=0\; & \text{on}\;\partial \Omega_{N}\times (t_{0},T), \end{array}\right.$$

endowed with the initial condition $u=u_{0}$ in $\Omega \times \{t_{0}\}$, where $\partial \Omega_{D}\cup \partial \Omega_{N}=\partial \Omega$, $\partial \Omega_{D}\cap \partial \Omega_{N}=\emptyset$, ν is the kinematic viscosity, and 𝕌 and ℚ are suitable Hilbert function spaces. The functions $u_{D}$ and $u_{0}$ are given.

The flow regime is defined by the Reynolds number

(2) $$Re≐\frac{UL}{\nu },$$

where *U* and *L* represent the characteristic velocity and length scales of the system, respectively. When the Reynolds number is large, the inertial forces dominate the viscous forces; this setting is generally referred to as the convection‐dominated regime. As explained in the introduction, it is well known that in the convection‐dominated regime standard spatial discretizations yield spurious numerical oscillations in under‐resolved and marginally‐resolved numerical simulations. In our numerical investigations in Sections 3.4, 3.5, and 4, we consider marginally‐resolved simulations. To alleviate the spurious numerical oscillations of standard spatial discretizations, we equip the FOM with the evolve‐filter‐relax (EFR) algorithm. This strategy has been exploited with standard numerical discretization techniques, ranging from FE to SE to FV methods: see, for example, References 5, 6, 7, 8, 9, 10, 11, 12, 13. In this article, we use the FE method and a backward differentiation formula of order 1 (BDF1) for the space and time discretization, respectively.

In what follows, we denote the semidiscrete FE velocity and pressure with $u\in {𝕌}^{N_{h}^{u}}$ and $p\in {\mathbb{Q}}^{N_{h}^{p}}$, respectively, where $N_{h}^{u}$ and $N_{h}^{p}$ are the corresponding numbers of degrees of freedom. We denote the time step with Δt. Let $t_{n}=t_{0}+n\Delta t$ for $n=0,\ldots ,N_{T}$, and $T=t_{0}+N_{T}\Delta t$. We denote with $y^{n}$ the approximation of a generic quantity *y* at the time $t^{n}$. The EFR algorithm at the time $t^{n+1}$ yields:

$$\begin{array}{llll} & \text{(I)}\;\text{Evolve}:\;\left\{\begin{array}{cc} \frac{w^{n+1}-u^{n}}{\Delta t}+(w^{n+1}\cdot \nabla )w^{n+1}-\nu \Delta w^{n+1}+\nabla p^{n+1}=0\; & \text{in}\;\Omega \times \{t_{n+1}\},\\ \nabla \cdot w^{n+1}=0\; & \text{in}\;\Omega \times \{t_{n+1}\}, \end{array}\right.\; & & \\ & \text{(II)}\;\text{Filter:}\;\left\{\begin{array}{cc} -\;2\delta^{2}\;\Delta {\bar{w}}^{n+1}+{\bar{w}}^{n+1}=w^{n+1}\; & \text{in}\;\Omega \times \{t_{n+1}\},\\ {\bar{w}}^{n+1}=u_{D}^{n+1}\; & \text{on}\;\partial \Omega_{D}\times \{t_{n+1}\},\\ \frac{\partial {\bar{w}}^{n+1}}{\partial n}=0\; & \text{on}\;\partial \Omega_{N}\times \{t_{n+1}\}. \end{array}\right.\; & & \\ & \text{(III)}\;\text{Relax:}\;u^{n+1}=(1-\chi )\;w^{n+1}+\chi \;{\bar{w}}^{n+1}\;,\; & & \end{array}$$

where χ∈[0,1] is a relaxation parameter. Here w is the evolved velocity and $\bar{w}$ is the filtered velocity. We note that using $w^{n+1}=u^{n+1}$ in step (I) (i.e., the evolve step) is equivalent to solving the NSE. In step (II), we use a *differential filter* (DF) with an explicit length scale, δ, which is the *filtering radius* (i.e., the radius of the neighborhood from which the spatial filter extracts information). The success of DFs is due to several appealing properties.^52^ For example, the DF leverages an elliptic operator and acts as a spatial filter by eliminating the small scales (i.e., high frequencies) from the input data. Step (III) is a relaxation step in which the EFR velocity approximation at the new time step is defined as a linear combination of the approximations in Step (I) and Step (II). The relaxation parameter χ diminishes the magnitude of the numerical diffusion^7^, ^12^, ^15^ and increases the accuracy; see, for example, the numerical results in Reference 14 and the theoretical results in Reference 15. The scaling χ∼Δt is commonly used.^15^ In References 10 and 14, however, the authors provide heuristic formulas that advocate higher values.

## 3 MODEL REDUCTION WITH RESPECT TO TIME

In this section, we focus on our POD‐Galerkin ROM framework for model reduction with respect to time. In Section 3.1, we give a brief description of the POD algorithm.^19^, ^25^, ^53^ Then, in Sections 3.2 and 3.3, we describe the two different ROM algorithms proposed, that is, EFR‐noEFR and EFR‐EFR. Finally, in Sections 3.4 and 3.5, we report and discuss some numerical experiments. All the ROM computations are performed with RBniCS,^54^ which is a FEniCS‐based^55^ library.

### 3.1 The POD algorithm

The basic idea of ROMs is to build a low‐dimensional framework where the problem at hand can be solved more efficiently than the FOM. To this end, assume that we have two bases, ${\left\{\varphi_{j}\right\}}_{j=1}^{r}$ and ${\left\{\psi_{j}\right\}}_{j=1}^{r}$, for the reduced velocity and pressure spaces ${𝕌}^{r}$ and ${\mathbb{Q}}^{r}$, respectively, so that

(3) $$u_{r}≐u_{r}(x,t)=\overset{r}{\underset{j=1}{\sum }}a_{j}^{u}(t)\varphi_{j}(x)\;\text{and}\;p_{r}≐p_{r}(x,t)=\overset{r}{\underset{j=1}{\sum }}a_{j}^{p}(t)\psi_{j}(x),$$

where ${\left\{a_{j}^{u}(t)\right\}}_{j=1}^{r}$ and ${\left\{a_{j}^{p}(t)\right\}}_{j=1}^{r}$ are the sought time‐varying coefficients.^56^ The bases are linear combinations of the snapshots, that is, FOM solutions computed at properly chosen time instances, ${\left\{u_{i}\right\}}_{i=1}^{N_{u}}\subseteq {\left\{u^{k}\right\}}_{k=1}^{N_{T}}$ and ${\left\{p_{i}\right\}}_{i=1}^{N_{p}}\subseteq {\left\{p^{k}\right\}}_{k=1}^{N_{T}}$, where $N_{u}$ and $N_{p}$ denote the number of snapshots for velocity and pressure, respectively. We utilize the EFR at the FOM level to generate the snapshots for both the EFR‐noEFR and the EFR‐EFR strategies. We employ the POD algorithm^19^, ^25^, ^53^ to compress the snapshot information and to build the reduced spaces.

It is well known that, in a standard NSE setting, the POD may be combined with a *supremizer stabilization* for the reduced velocity space in order to guarantee the well‐posedness of the system. We emphasize that the main role of the supremizers is to avoid spurious reduced pressure modes. To tackle the convection‐dominated, marginally‐resolved regime, different approaches are needed. The supremizer stabilization proposed in Reference 24 relies on a supremizer operator $S:{\mathbb{Q}}^{N_{h}^{p}}\to {𝕌}^{N_{h}^{u}}$ defined as

(4) $${\left(S(p),\tau \right)}_{𝕌}=(p,\nabla \cdot \tau ),\;\forall \tau \in {𝕌}^{N_{h}^{u}}.$$

Then, the considered reduced velocity space is

(5) $${𝕌}^{r_{us}}≐\text{POD}({\left\{u_{i}\right\}}_{i=1}^{N_{u}})⊕\text{POD}({\left\{S(p_{i})\right\}}_{i=1}^{N_{u}}),$$

where ${\left\{p_{i}\right\}}_{i=1}^{N_{u}}$ in (5) are the pressure snapshots related to the velocity snapshots, that is, derived from the solution ${\left(u_{i},p_{i}\right)}_{i=1}^{N_{u}}$. However, a standard POD procedure is applied to ${\left\{p_{i}\right\}}_{i=1}^{N_{p}}$:

$${\mathbb{Q}}^{r_{p}}≐\text{POD}({\left\{p_{i}\right\}}_{i=1}^{N_{p}}),$$

where only the first $r_{p}$ POD eigenpairs are retained to build the bases. The supremizer technique leads to a reduced velocity space of dimension $r_{us}=r_{u}+r_{s}$. We denote the enriched reduced velocity dimension with $r_{us}$, and consider ${\left\{\varphi_{j}\right\}}_{j=1}^{r_{us}}$ as the enlarged velocity‐supremizer basis. We define the pressure basis as ${\left\{\psi_{k}\right\}}_{k=1}^{r_{p}}$.

### 3.2 EFR‐noEFR

The EFR‐noEFR consists of the Galerkin projection of the NSE on the reduced space, which leads to the solution of the following system: at the time $t^{n+1}$, find the pair $\left(u_{r}^{n+1},p_{r}^{n+1}\right)$ that solves

(6) $$\left\{\begin{array}{c} \left(\frac{u_{r}^{n+1}-u_{r}^{n}}{\Delta t},\varphi_{i}\right)+\nu \;(\nabla u_{r}^{n+1},\nabla \varphi_{i})+((u_{r}^{n+1}\cdot \nabla )u_{r}^{n+1},\varphi_{i})-(p_{r}^{n+1},\nabla \cdot \varphi_{i})=0,\\ (\nabla \cdot u_{r}^{n+1},\psi_{k})=0, \end{array}\right.$$

for all $i=1,\ldots ,r_{us},$ and $j=1,\ldots ,r_{p}$. Algebraically, we are looking for the (n+1)‐st solution of

(7) $$\left\{\begin{array}{c} \frac{1}{\Delta t}M(u^{n+1}-u^{n})+\nu Ku^{n+1}+C(u^{n+1})u^{n+1}-B^{T}p^{n+1}=0,\\ Bu^{n+1}=0, \end{array}\right.$$

where $u^{n+1}\in {\mathbb{R}}^{r_{us}}$ and $p^{n+1}\in {\mathbb{R}}^{r_{p}}$ are the vectors of the reduced coefficients of (3) and represent the unknowns of the problem, M is the reduced velocity space mass matrix, K the reduced stiffness matrix, that is,

(8) $$M_{ij}≐(\varphi_{i},\varphi_{j})\;\text{and}\;K_{ij}≐(\nabla \varphi_{i},\nabla \varphi_{j}),$$

while

(9) $$C{\left(u^{n+1}\right)}_{ij}≐((u^{n+1}\cdot \nabla )\varphi_{i},\varphi_{j})\;\text{and}\;B_{ij}≐(\nabla \cdot \varphi_{i},\psi_{j}).$$

In Algorithm 1, we present the pseudocode for EFR‐noEFR: the POD bases are extracted from EFR solutions, the supremizer enrichment is performed, and the standard NSE are projected on the reduced spaces.

Algorithm 1 **Pseudocode for EFR‐noEFR**

1

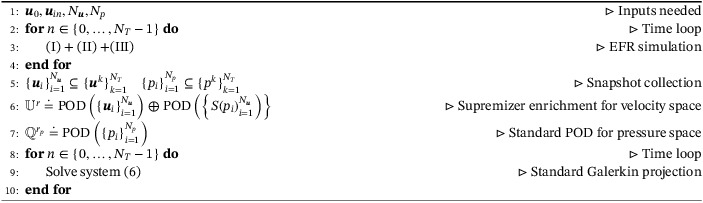

### 3.3 EFR‐EFR

In the EFR‐EFR, we apply a double stabilization. Specifically, we employ the EFR algorithm not only at the FOM level, but also at the ROM level. Indeed, after the POD modes are built from the EFR snapshots, we apply the EFR steps (I), (II), and (III) in a reduced setting, as specified in Algorithm 2:

$$\begin{array}{llll} & {\text{(I)}}_{r}\;\left\{\begin{array}{c} \left(\frac{w_{r}^{n+1}-u_{r}^{n}}{\Delta t},\varphi_{i}\right)+\nu \;(\nabla w_{r}^{n+1},\nabla \varphi_{i})+((w_{r}^{n+1}\cdot \nabla )w_{r}^{n+1},\varphi_{i})-(p_{r}^{n+1},\nabla \cdot \varphi_{k})=0,\\ (\nabla \cdot w_{r}^{n+1},\psi_{i})=0, \end{array}\right.\; & & \\ & {\text{(II)}}_{r}\;2\delta^{2}\;(\nabla {\bar{w}}_{r}^{n+1},\nabla \varphi_{i})+({\bar{w}}_{r}^{n+1},\varphi_{i})=(w_{r}^{n+1},\varphi_{i}),\; & & \\ & \; & & \\ & {\text{(III)}}_{r}\;u_{r}^{n+1}=(1-\chi )\;w_{r}^{n+1}+\chi \;{\bar{w}}_{r}^{n+1}.\; & & \end{array}$$

As we did in (3), we expand the reduced variables $w_{r}$ and ${\bar{w}}_{r}$ of ${𝕌}^{r}$ as

(10) $$w_{r}≐w_{r}(x,t)=\overset{r}{\underset{j=1}{\sum }}a_{j}^{w}(t)\varphi_{j}(x)\;\text{and}\;{\bar{w}}_{r}≐{\bar{w}}_{r}(x,t)=\overset{r}{\underset{j=1}{\sum }}a_{j}^{\bar{w}}(t)\varphi_{j}(x).$$

Thus, at the time instance $t^{n+1}$, we solve the following system:

(11) $$\left\{\begin{array}{c} \frac{1}{\Delta t}M(w^{n+1}-u^{n})+\nu Kw^{n+1}+C(w^{n+1})w^{n+1}-B^{T}p^{n+1}=0,\\ Bw^{n+1}=0,\\ 2\delta^{2}K{\bar{w}}^{n+1}+M{\bar{w}}^{n+1}=Mw^{n+1},\\ u^{n+1}=(1-\chi )w^{n+1}+\chi {\bar{w}}^{n+1}, \end{array}\right.$$

where $w^{k+1}\in {\mathbb{R}}^{r_{us}}$ and ${\bar{w}}^{k+1}\in {\mathbb{R}}^{r_{us}}$ are the unknown reduced coefficient vectors of the evolved and filtered velocity fields, respectively, as defined in (10). All the matrices in (11) have been defined in (8) and (9). Although the DF has been widely used in a ROM framework,^33^, ^35^, ^41^, ^57^, ^58^, ^59^ to the best of our knowledge, the analysis of a *Relax* step is still limited.^29^ We stress that, at the ROM level, the computational effort of the DF filter (II)_*r*_ and the relaxation step (III)_*r*_ is negligible and thus the costs of the EFR‐noEFR and EFR‐EFR will be comparable. Moreover, the ROM model is consistent with respect to the choice of δ and χ, which are the same as those used in the FOM model.

For the sake of clarity, in Table 1, we report all the acronyms that we use, together with the corresponding equations or algorithms. We also note that, in what follows, we make no distinction between the EFR and FOM simulation, since in the FOM numerical results the EFR strategy is always performed.

**TABLE 1:** Acronyms

Acronym	Equation or algorithm	Offline stabilization	Online stabilization
EFR (or FOM)	(I) + (II) + (III)	✓	
EFR‐noEFR	Algorithm 1	✓	
EFR‐EFR	Algorithm 2	✓	✓
EFR‐noEFR and n‐POD	Algorithm 3	✓	
EFR‐EFR and n‐POD	Algorithm 4	✓	✓

Remark 2 We remark that, for the NSE, the online phase still depends on the FOM dimension and this affects the EFR‐noEFR and EFR‐EFR performances in terms of computational time. For this reason we are not presenting a comparative analysis between the FOM and the ROM solutions with respect to the computational costs. To overcome this issue, hyper‐reduction techniques, such as the empirical interpolation method (EIM), may be employed, see, for example, References 19 (chapter 5) or 60. However, this goes beyond the scope of the present work.

Algorithm 2 **Pseudocode for EFR‐EFR**

1

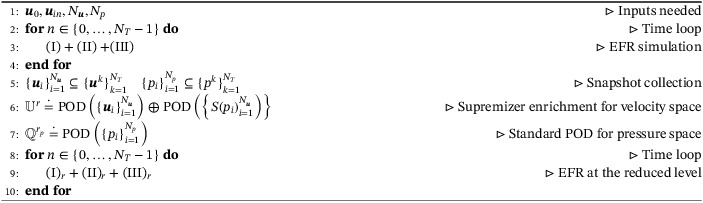

### 3.4 Numerical results: Reconstructive regime

In this section, we analyze and compare the performances of EFR‐noEFR (see Algorithm 1) and EFR‐EFR (see Algorithm 2). The goal is to investigate the FOM‐ROM consistency for the EFR stabilization algorithm. We consider an incompressible 2D flow past a cylinder at time‐dependent Reynolds number 0≤Re≤100. This benchmark has been thoroughly studied at full order level^37^, ^38^, ^41^ (Figure1).

**IGURE 1:**
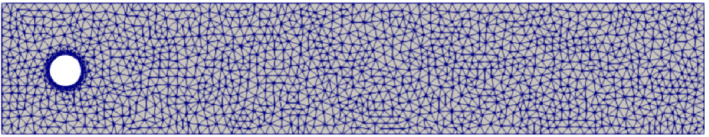
F The FE mesh

**FIGURE 2:**
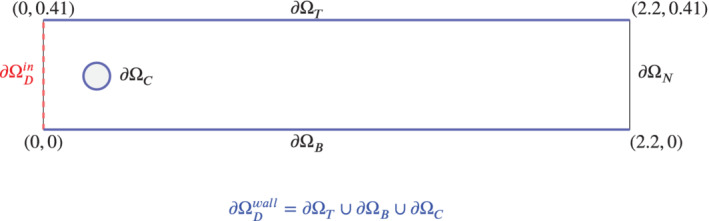
The computational domain, Ω. $\partial \Omega_{D}≐\partial \Omega_{D}^{in}\cup \partial \Omega_{D}^{wall}$, where the inlet boundary $\partial \Omega_{D}^{in}$ is represented by a dashed red line and the no‐slip boundaries by a solid blue line

We consider the motion of an incompressible flow in a domain $\Omega ≐\{[0,2.2]\times [0,0.41]\}\setminus \{(x,y)\in {\mathbb{R}}^{2}\;\text{such that}\;{\left(x-0.2\right)}^{2}+{\left(y-0.2\right)}^{2}-0.05^{2}=0\}$, which is depicted in Figure 2. We set $\nu =10^{-3}$ and use no‐slip boundary conditions on $\partial \Omega_{D}^{wall}$, representing the union of the lower and upper walls of the channel, and the cylinder wall (solid blue boundary in Figure 2), with a time varying inlet velocity profile $u_{in}$ on $\partial \Omega_{D}^{in}$ (red dashed line in Figure 2). The prescribed inlet condition is given by

(12) $$u_{in}≐\left(\frac{0.6}{0.41^{2}}\sin(\pi t/8)y(0.41-y),0\right).$$

Furthermore, on $\partial \Omega_{N}$ (black line in Figure 2) we employ homogeneous Neumann conditions. The value of the initial condition $u_{0}$ is (0,0). The time‐dependent inlet velocity leads to a Reynolds number that varies in time,^38^ with 0≤Re≤100. We perform our tests on a triangular mesh with $h_{\min}=4.46\cdot 10^{-3}$ and $h_{\max}=4.02\cdot 10^{-2}$ (Figure 1). We employ the Taylor‐Hood ${\mathbb{P}}^{2}-{\mathbb{P}}^{1}$ FE pair for velocity and pressure, respectively, and this leads to a FE space of dimension $N_{h}≐N_{h}^{u}+N_{h}^{p}=14053$.

Remark 3 We note that the mesh that we use in our numerical investigation does not feature the level of refinement required by a direct numerical simulation, which is about 100k degrees of freedom.^37^ Therefore, although the Reynolds number is not high, the computational setting is still relatively challenging for the FOM and ROM simulations. For the EFR validation at the FOM level performed by investigating this benchmark on coarse meshes, the reader is referred to References 10, 14, 61.

For both the FOM and the ROM simulations, we set $\Delta t=4\cdot 10^{-4}$ and $\delta =L\cdot Re^{-3/4}=0.0032$ as in Reference 41 (i.e., we set the filter radius to the Kolmogorov scale^62^, ^63^).

Remark 4 The value of δ is still an arguable choice in CFD applications. For unstructured meshes, a common choice is to set the filtering radius as the $h_{\min}$. Indeed, $\delta =h_{\min}$ avoids an excessive diffusion action of system over the elements of the mesh.^14^ However, in the following experiments, we used the Kolmogorov scale $L\cdot Re^{-\frac{3}{4}}$ to be consistent with the parameters of Reference 10. Moreover, we underline that $L\cdot Re^{-\frac{3}{4}}∼h_{\min}$, thus the choice is reasonable.

We define the $L^{2}$ relative errors for the velocity and the pressure fields, respectively, as

(13) $$E_{u}(t)≐\frac{\|u(t)-u_{r}(t){\|}_{L^{2}(\Omega )}}{\|u(t){\|}_{L^{2}(\Omega )}}\;\text{and}\;E_{p}(t)≐\frac{\|p(t)-p_{r}(t){\|}_{L^{2}(\Omega )}}{\|p(t){\|}_{L^{2}(\Omega )}}.$$

Furthermore, we test the ROM accuracy by using the drag coefficient

(14) $$C_{D}(t)≐\frac{2}{U^{2}L}\int_{\partial \Omega_{C}}((2\nu \nabla u-pI)\cdot n_{C})\cdot t_{C}\;ds,$$

and the lift coefficient

(15) $$C_{L}(t)≐\frac{2}{U^{2}L}\int_{\partial \Omega_{C}}((2\nu \nabla u-pI)\cdot n_{C})\cdot n_{C}\;ds,$$

where $n_{C}$ and $t_{C}$ are the normal and tangential unit vectors to the cylinder boundary $\partial \Omega_{C}$ (see Figure 2), respectively. Specifically, we compute the $L^{2}$‐errors of the force coefficients:

(16) $${\bar{E}}_{C_{D}}≐\frac{\|C_{D}-{\bar{C}}_{D}{\|}_{L^{2}(t_{0},T)}}{\|C_{D}{\|}_{L^{2}(t_{0},T)}}\;\text{and}\;{Ê}_{C_{D}}≐\frac{\|C_{D}-{Ĉ}_{D}{\|}_{L^{2}(t_{0},T)}}{\|C_{D}{\|}_{L^{2}(t_{0},T)}},$$

(17) $${\bar{E}}_{C_{L}}≐\frac{\|C_{L}-{\bar{C}}_{L}{\|}_{L^{2}(t_{0},T)}}{\|C_{L}{\|}_{L^{2}(t_{0},T)}}\;\text{and}\;{Ê}_{C_{L}}≐\frac{\|C_{L}-{Ĉ}_{L}{\|}_{L^{2}(t_{0},T)}}{\|C_{L}{\|}_{L^{2}(t_{0},T)}}.$$

We denote the EFR‐noEFR drag and lift coefficients as ${\bar{C}}_{D}(t)$ and ${\bar{C}}_{L}(t)$, respectively. Similarly, we denote the EFR‐EFR drag and lift coefficients as ${Ĉ}_{D}(t)$ and ${Ĉ}_{L}(t)$.

Remark 5 At the FOM level, one has to tackle the issue of preserving the incompressibility constraint when applying the DF (II). Indeed, while the DF preserves the incompressibility under periodic boundary conditions, it does not preserve the incompressibility under no‐slip boundary conditions.^15^, ^64^, ^65^ In our specific test case, this might translate in unacceptable divergence values near the cylinder boundary, $\partial \Omega_{C}$. In References 15 and 64, a Stokes differential filter is proposed as a solution to recover the mass conservation at the DF level. However, as underlined by the authors, this is a more expensive filtering operation. We decided to address this problem by exploiting another technique, easier to implement and which gave us acceptable divergence values: a div‐grad stabilization that penalizes the violation of the incompressibility constraint.^66^, ^67^ Namely, in the DF equations, we added a term of the form

(18) $$\gamma \nabla (\nabla \cdot {\bar{w}}^{n+1}),$$

with γ=100, as used in Reference 66. The reader interested in an overview of grad‐div stabilization and the choice of γ may refer to References 67, 68, 69.

In our investigation, we present numerical experiments where the relaxation is considered and others where it is not. When χ≠1, we do not use the grad‐div stabilization at the FOM level. The rationale for our choice is the following. When we utilize the Relax step (III), it expresses the velocity approximation as a convex combination of the intermediate velocity approximation obtained in the Evolve step (I) and the filtered velocity approximation obtained in the Filter step (II). Since the Evolve step (I) enforces the incompressibility constraint in the intermediate velocity approximation, the velocity approximation in the Relax step (III) displays low divergence values and the grad‐div stabilization is no longer needed in this scenario.

We also note that other techniques may be employed to achieve divergence‐free snapshots,^67^ such as the filters described in Reference 57.

**Experiment 1**.

As a first step in the comparison of EFR‐noEFR and EFR‐EFR, we consider the EF stabilization strategy both at the FOM level and at the ROM level. That is, we discard the *Relax* step (i.e., we consider χ=1) both for EFR‐noEFR and for EFR‐EFR. We note that, in a FOM (and, consequently, in a ROM) setting, the EF algorithm is overdiffusive and high frequency modes are completely damped (see, e.g., Reference 10).

**FIGURE 3:**
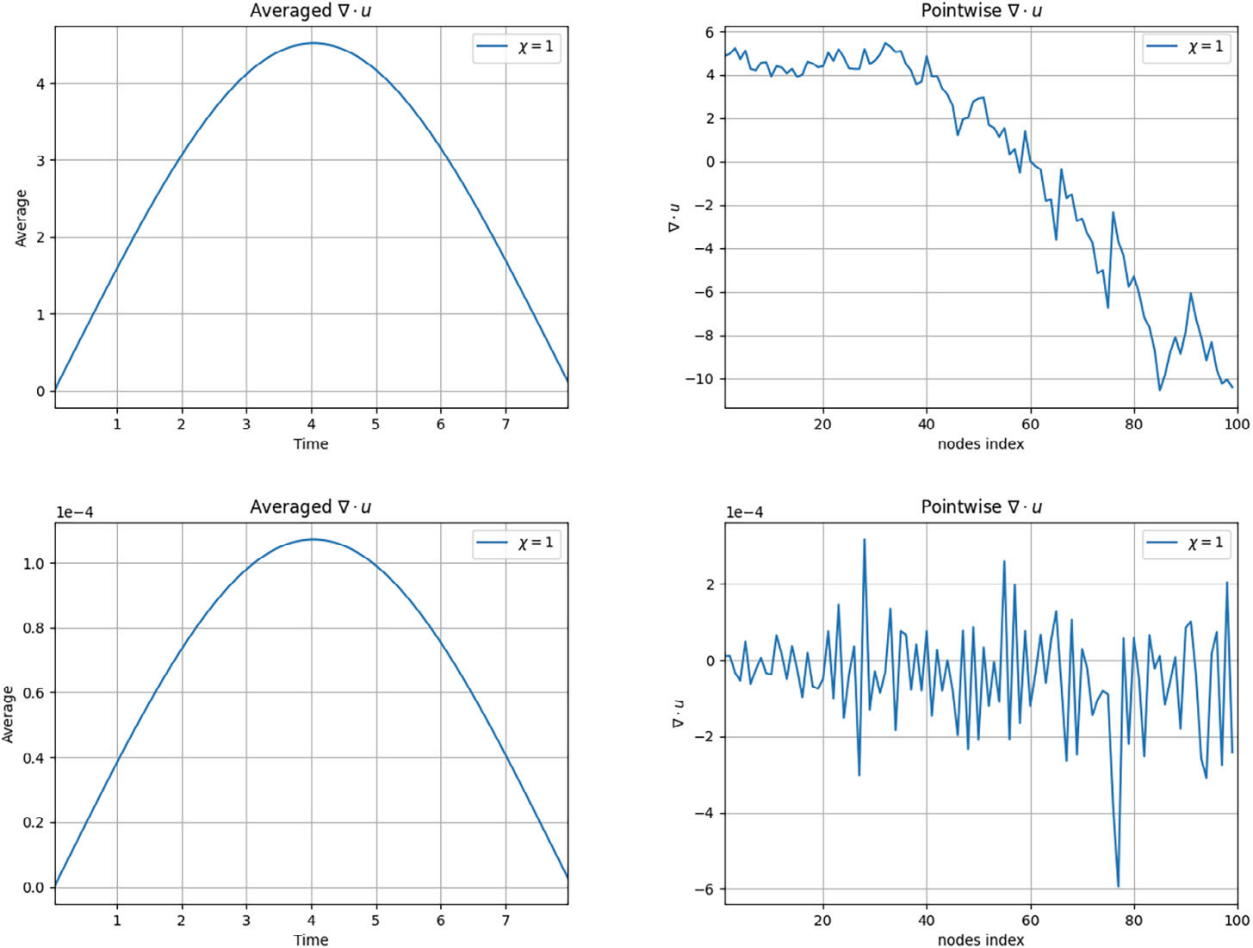
Experiment 1: δ=0.0032, χ=1, full order EFR results. *Top left and top right*: Av(t) and divergence nodal values over the cylinder without grad‐div stabilization. *Bottom left and bottom right*: Av(t) and divergence nodal values over the cylinder with grad‐div stabilization

As already specified in Remark 5, we apply the grad‐div stabilization at a FOM level to enforce the incompressibility constraint along the cylinder boundary. The improvements with respect to the incompressibility of the flow are displayed in Figure 3. We denote with ${\left(x_{i}^{c},y_{i}^{c}\right)}_{i=1}^{N_{c}}$ the mesh nodal coordinates related to the cylinder boundary $\partial \Omega_{C}$. In our case, $N_{c}=100$. We plot

(19) $$Av(t)=\frac{1}{N_{c}}\overset{N_{c}}{\underset{i}{\sum }}|\nabla \cdot u(x_{i},y_{i})|,$$

that is, the averaged absolute value of the divergence over the nodal coordinates at time *t*, and the nodal values $\nabla \cdot u(x_{i},y_{i})$, for 1≤i≤100 for t=4, which is the time instance with the worst behavior with respect to the incompressibility constraint violation (as can be seen in the left panel of Figure 3). As expected, the use of the grad‐div stabilization allows us to reach much smaller divergence values in time. Indeed, from values of order O(1), thanks to the grad‐div stabilization term, we obtain values of order $O(10^{-4})$.

We note that we utilize the grad‐div stabilization at the FOM level, but not at the ROM level. Thanks to the grad‐div stabilization, the velocity snapshots display acceptable divergence values. Thus, the divergence values of the ROM velocity approximations are relatively low and the grad‐div stabilization is not needed at the ROM level, that is, FOM and ROM are not consistent with respect to grad‐div stabilization. Moreover, in our specific case, using the grad‐div stabilization at the ROM level with the same parameters as those used at the FOM level leads to an over‐diffusive reconstruction of the aerodynamics coefficients at the ROM level. For the application of the grad‐div stabilization at the ROM level, the interested reader is referred to Reference 70.

**FIGURE 4:**
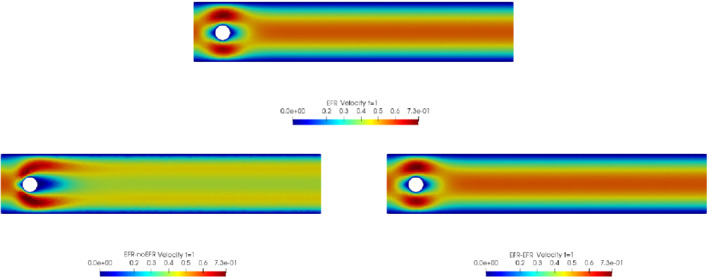
Experiment 1: δ=0.0032, χ=1, r=2 and t=1. *Top*: full order EFR velocity magnitude. *Bottom left*: reduced EFR‐noEFR velocity magnitude. *Bottom right*: reduced EFR‐EFR velocity magnitude

**FIGURE 5:**
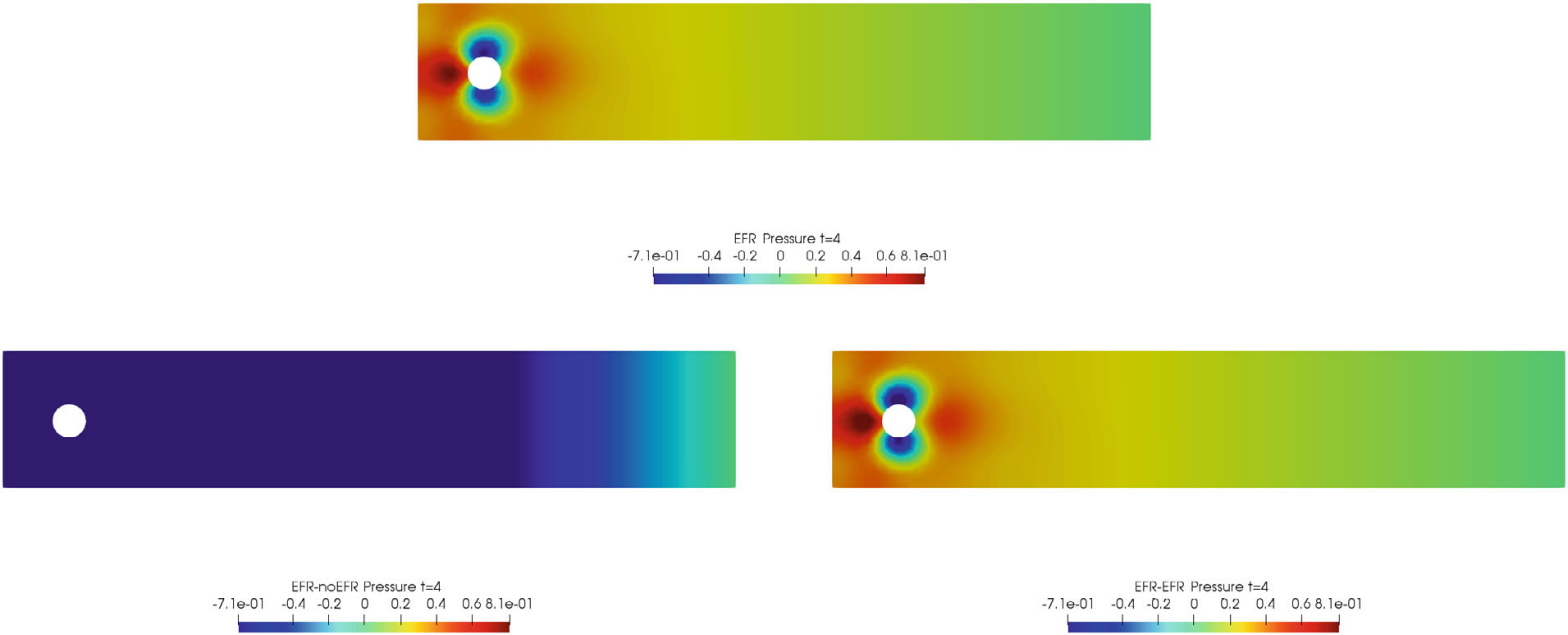
Experiment 1: δ=0.0032, χ=1, r=2 and t=4. *Top*: full order EFR pressure field. *Bottom left*: reduced EFR‐noEFR pressure field. *Bottom right*: reduced EFR‐EFR pressure field

**FIGURE 6:**
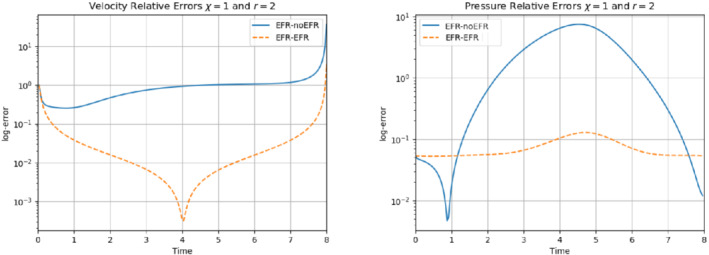
Experiment 1: δ=0.0032, χ=1 and r=2. *Left*: comparison of relative log‐errors over time of the velocity profiles: EFR full order versus EFR‐noEFR solutions and EFR full order versus EFR‐EFR solutions, represented by solid blue and dashed orange lines, respectively. *Right*: analogous representation for the relative log‐errors over time of the pressure profiles

**FIGURE 7:**
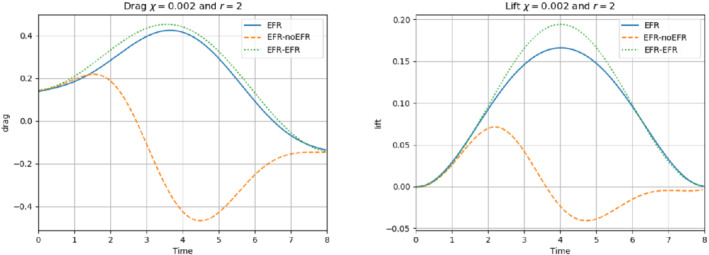
Experiment 1: δ=0.0032, χ=1 and r=2. *Left*: $C_{D}(t)$ comparison over time. *Right*: $C_{L}(t)$ comparison over time

We collect $N_{u}=N_{p}=200$ snapshots for both velocity and pressure with an equally spaced grid in the time interval $\left[t_{0},T]=[0,8\right]$. After the POD procedure, we retain the first 2 modes for both velocity and pressure, representing 99.9% of the snapshot energy. Here, for the sake of notation, we use $r≐r_{u}=r_{s}=r_{p}=2$. We show representative solutions of velocity for t=1 and pressure for t=4 in Figures 4 and 5, respectively. It is clear that the EFR‐noEFR is not able to reconstruct the solution provided by the FOM, while the EFR‐EFR leads to very accurate results for both the fields. To allow an easy comparison, we plot the velocity and pressure fields on the FOM scale, that is, [0,0.7] and [−0.71,0.61], respectively. The relative log‐error temporal trend, displayed in Figure 6, confirms this conclusion: it shows how the EFR‐EFR yields more accurate results for both velocity and pressure, reaching values around $10^{-3}$ for the velocity, and reducing the error by two orders of magnitude with respect to the EFR‐noEFR for both variables. The comparison between FOM and ROM aerodynamic coefficients over time is reported in Figure 7. The coefficients are well recovered by the EFR‐EFR, while the EFR‐noEFR is not able to accurately approximate them. Indeed, the relative errors (16) and (17) have the following values: ${\bar{E}}_{C_{D}}=1.02$, ${Ê}_{C_{D}}=0.13$, and ${\bar{E}}_{C_{L}}=1.69$, ${Ê}_{C_{L}}=0.11$. The advantage of using the EFR‐EFR is remarkable, since we are reducing the relative $L^{2}$‐errors of the force coefficients by an order of magnitude. Table 2 lists the maximum, minimum, and average relative errors for the velocity and pressure fields in the EFR‐noEFR and EFR‐EFR settings. Overall, the results in Table 2 are consistent with the plots in Figure 6. With respect to the velocity approximation, the EFR‐EFR is significantly more accurate than the EFR‐noEFR. The maximum, minimum, and average relative errors are at least one order of magnitude lower for the EFR‐EFR than for the EFR‐noEFR. With respect to the pressure approximation, the EFR‐EFR is again more accurate than the EFR‐noEFR, but the improvement is not as dramatic as for the velocity approximation.

**TABLE 2:** Experiment 1: δ=0.0032, χ=1 and r=2

	EFR‐noEFR			EFR‐EFR		
	Maximum	Minimum	Average	Maximum	Minimum	Average
$E_{u}(t)$	3.721e+1	2.533e‐1	1.115e+0	3.298e+0	3.145e‐4	6.252e‐2
$E_{p}(t)$	7.450e+0	3.431e‐3	2.306e+0	1.620e‐1	2.877e‐2	1.701e‐2

*Note*: Maximum, minimum, and average relative error over the considered time interval for velocity and pressure fields.

**Experiment 2**. The next step in our comparison of EFR‐noEFR and EFR‐EFR is the numerical investigation of the EFR stabilization strategy both at the FOM level and at the ROM level. Specifically, we use χ=5·Δt=0.002 in the *Relax* step for both the EFR‐noEFR and the EFR‐EFR. This choice limits the amount of dissipation introduced by the DF in the *Filter* step of the EFR algorithm, and yields a more challenging test problem than Experiment 1 for both the EFR‐noEFR and the EFR‐EFR. Specifically, Experiments 1 and 2 share the same computational setting except that for Experiment 1 no relaxation is performed. Indeed, in Experiment 1 we use χ=1, and thus

(20) $$u_{r}^{n+1}=(1-\chi )\;w_{r}^{n+1}+\chi \;{\bar{w}}_{r}^{n+1}={\bar{w}}_{r}^{n+1}.$$

The relationship in (20) shows that, in Experiment 1, the final velocity coincides with the filtered velocity. This setting is overly diffusive and, as a result, the vortices that appear in Experiment 2 are totally damped in Experiment 1. We also note that, in contrast with Experiment 1, in Experiment 2 we do not use the grad‐div stabilization at the FOM level (see Remark 5). Indeed, in Experiment 2, the divergence values are $O(10^{-2})$ in the worst case scenario. Thus, the grad‐div stabilization is no longer needed and the FOM and ROM are consistent with respect to the grad‐div stabilization.

Remark 6 As already noted in Section 2, χ∼Δt is a common choice used in the literature for academic benchmarks (see, e.g., Reference 15). However, in Reference 14 the authors propose the scaling χ=cΔt. Thus, we choose c=5, that is, a higher χ value, which introduces a larger amount of dissipation. This setting can be of interest in more realistic applications.^10^, ^14^

We construct and test the EFR‐noEFR and EFR‐EFR on the time interval [4,8]. The rationale for our choice is that the flow dynamics is significantly more complex on the time interval [4,8] than on the time interval [0,4] (see Experiment 3).

**FIGURE 8:**
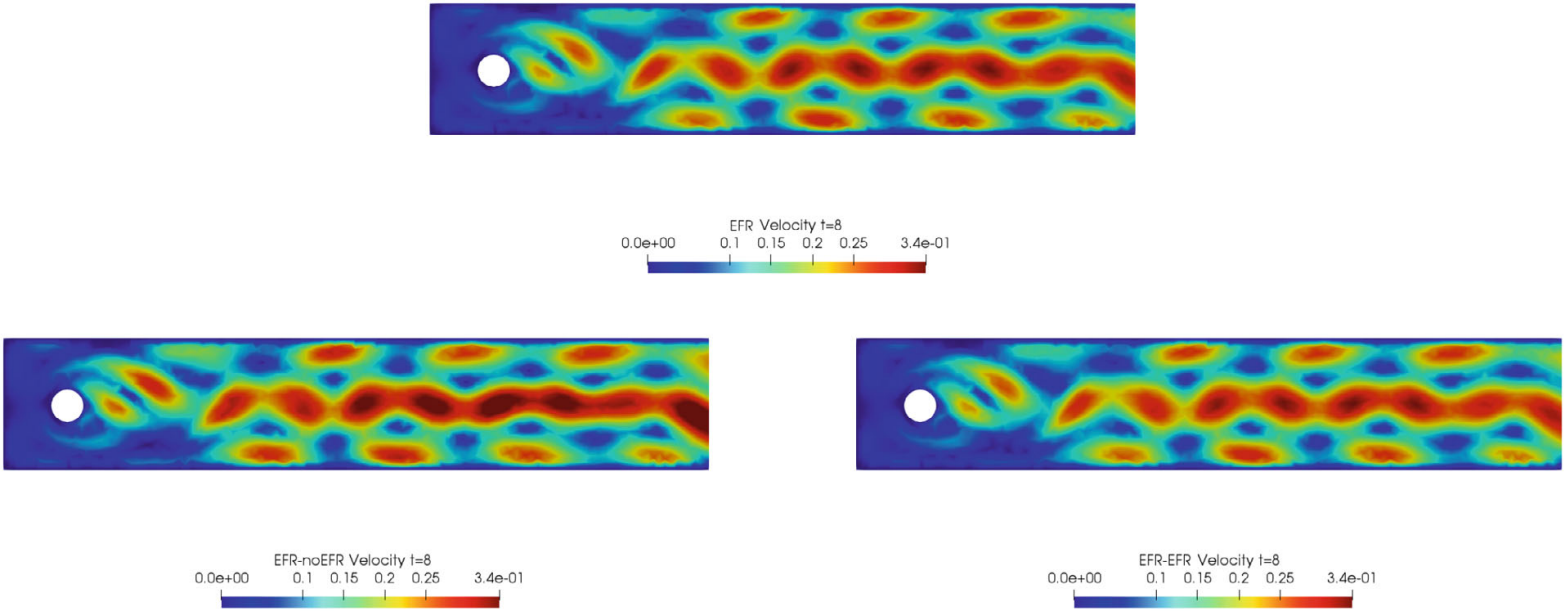
Experiment 2: δ=0.0032, χ=0.002, $r_{u}=43,r_{p}=r_{s}=8$ and t=8. Reconstruction for t∈[4,8]. *Top*: full order EFR velocity magnitude. *Bottom left*: reduced EFR‐noEFR velocity magnitude. *Bottom right*: reduced EFR‐EFR velocity magnitude

**FIGURE 9:**
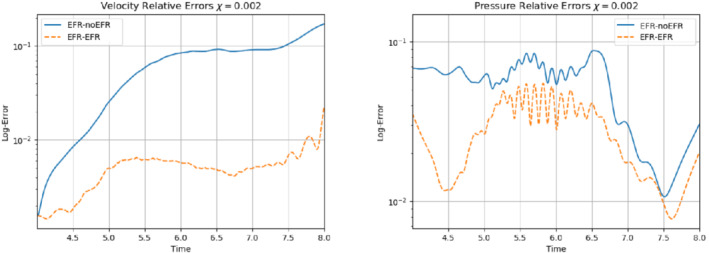
Experiment 2: δ=0.0032, χ=0.002 and $r_{u}=43,r_{p}=r_{s}=8$. Reconstruction for t∈[4,8]. *Left*: comparison of relative log‐errors over time for the velocity profiles: EFR‐noEFR (solid blue line) and EFR‐EFR (dashed orange line). *Right*: comparison of relative log‐errors over time for the pressure profiles: EFR‐noEFR (solid blue line) and EFR‐EFR (dashed orange line)

In order to approximate all the relevant features of the flow field, we increase the number of snapshots. Indeed, to build the EFR‐noEFR and EFR‐EFR, we collect $N_{u}=N_{p}=2000$ snapshots, which are equally spaced on the time interval [4,8]. To retain 99.9% of the snapshots energy, we employ the following numbers of POD basis functions to build the ROMs: $r_{u}=43$, $r_{s}=r_{p}=8$. We note that, with respect to Experiment 1, the system shows a slower decay of the eigenvalues and therefore more modes need to be used to construct the ROMs. The higher accuracy of EFR‐EFR is displayed in Figure 8: the EFR‐EFR solution perfectly matches the FOM solution, while the EFR‐noEFR solution is slightly different from the FOM solution. The relative log‐errors in Figure 9 (left) yield the same conclusions: the EFR‐EFR velocity errors are an order of magnitude lower than the EFR‐noEFR velocity errors. We also note that EFR‐EFR is more accurate than the EFR‐noEFR in approximating the pressure field, especially at the beginning and at the end of the time interval (see Figures 9 (right) and 10). The force coefficients $C_{D}(t)$ and $C_{L}(t)$ plotted in Figure 11 show that, while both EFR‐noEFR and EFR‐EFR are accurate, the latter is more accurate than the former. This is also illustrated by the $L^{2}$‐errors of the force coefficients: ${\bar{E}}_{C_{L}}=0.26$, ${Ê}_{C_{L}}=0.11$ and ${\bar{E}}_{C_{D}}=0.018$, ${Ê}_{C_{D}}=0.010$. The maximum, minimum, and average error values over time for the velocity and pressure fields, which are listed in Table 3, confirm that the EFR‐EFR is more accurate than the EFR‐noEFR. The improvement in the EFR‐EFR is also highlighted by the Pareto plot in Figure 12. Indeed, fixing $r_{p}=r_{s}=8$ and choosing $r_{u}=30,32,34,36,38,40,42,44,46,48,50$ shows that, over this range of $r_{u}$ values, the EFR‐EFR performs better than the EFR‐noEFR with respect to both the velocity and the pressure approximations. Indeed, the EFR‐EFR with $r_{u}=30$ yields a low relative error that the EFR‐noEFR cannot attain by increasing its $r_{u}$ value (and, consequently, its relative wall time).

**FIGURE 10:**
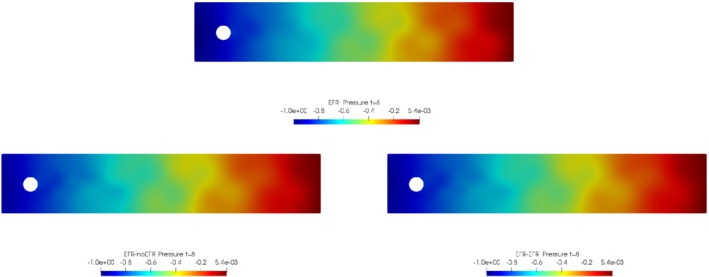
Experiment 2: δ=0.0032, χ=0.002, $r_{u}=43,r_{p}=r_{s}=8$, and t=8. Reconstruction for t∈[4,8]. *Top*: full order EFR pressure field. *Bottom left*: Reduced EFR‐noEFR pressure field. *Bottom right*: reduced EFR‐EFR pressure field

**FIGURE 11:**
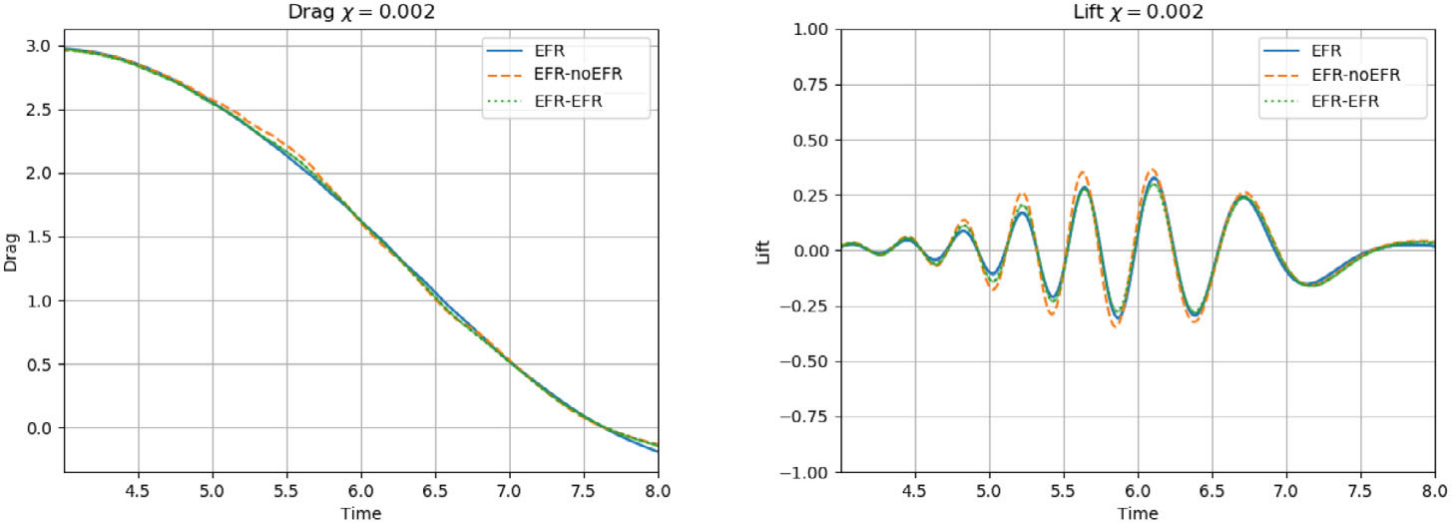
Experiment 2: δ=0.0032, χ=0.002, $r_{u}=43$, and $r_{p}=r_{s}=8$. Reconstruction for t∈[4,8]. *Left*: $C_{D}(t)$ comparison over time: all the approaches almost coincide. *Right*: $C_{L}(t)$ comparison over time: full order EFR and EFR‐EFR lift coincide (solid blue and dotted green lines)

**TABLE 3:** Experiment 2: δ=0.0032, χ=0.002, $r_{u}=43,r_{p}=r_{s}=8$

	EFR‐noEFR			EFR‐EFR		
	Maximum	Minimum	Average	Maximum	Minimum	Average
$E_{u}(t)$	1.718e‐1	1.561e‐3	6.781e‐2	2.267e‐2	1.446e‐3	5.175e‐3
$E_{p}(t)$	8.815e‐2	1.068e‐2	5.366e‐2	5.491e‐2	7.753e‐3	2.688e‐2

*Note*: Reconstruction for t∈[4,8]. Maximum, minimum, and average relative error over the considered time interval for velocity and pressure fields.

**FIGURE 12:**
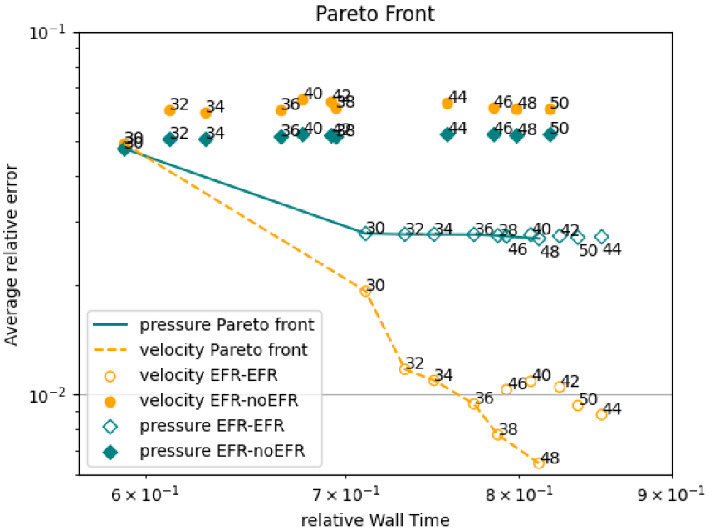
Experiment 2: δ=0.0032, χ=0.002 and $r_{u}=\{30,32,34,36,38,40,42,44,46,48,50\}$, $r_{p}=r_{s}=8$. Pareto plots for velocity (orange) and pressure (teal) fields: averaged relative error in time versus relative wall time for varying $r_{u}$

Overall, the numerical investigation in Experiment 2 yields the same conclusions as the numerical investigations in Experiment 1: the EFR‐EFR is more accurate than the EFR‐noEFR with respect to all the criteria used, that is, pointwise, average, maximum, and minimum velocity and pressure errors, lift and drag coefficient errors, and Pareto front. Thus, these numerical results suggest that the FOM‐ROM consistency is beneficial for the EFR stabilization strategy.

**Experiment 3**.

In this experiment, which is based on the computational setting in References 37, 38, 41, we investigate the EFR‐noEFR and EFR‐EFR models in a regime that is more challenging than the regime used in Experiment 2. Specifically, we consider a regime that, while still marginally‐resolved, employs a coarser resolution than Experiment 2 (i.e., relatively fewer snapshots and relatively fewer basis functions) and two different dynamical regimes (a laminar regime in the first half of the time interval, and a more complex regime in the second half). This investigation focuses on the reconstruction of the whole time interval [0,8] using $N_{u}=N_{p}=2000$ snapshots that are equally spaced in this time interval. Thus, we are using coarser resolution than the resolution used in Experiment 2, since we are employing the same number of snapshots as in Experiment 2 but consider a time interval that is twice as long as that used in Experiment 2. Furthermore, in the first half of the time interval the flow displays laminar dynamics, whereas in the second half it displays more complex dynamics (e.g., vortex shedding). We note that the mixed dynamics in Experiment 3 is more challenging to represent at the ROM level than the dynamics in Experiment 2 (i.e., in the time interval [4,8]). In order to retain 99.9% of the energy of the snapshots, we choose $r_{u}=47$ and $r_{p}=r_{s}=7$. We note that in Experiment 3 we utilize a similar number of basis functions as in Experiment 2. Since the dynamics in Experiment 3 is more challenging than the dynamics in Experiment 2, we conclude that the Experiment 3 ROM resolution is coarser than the Experiment 2 resolution.

**FIGURE 13:**
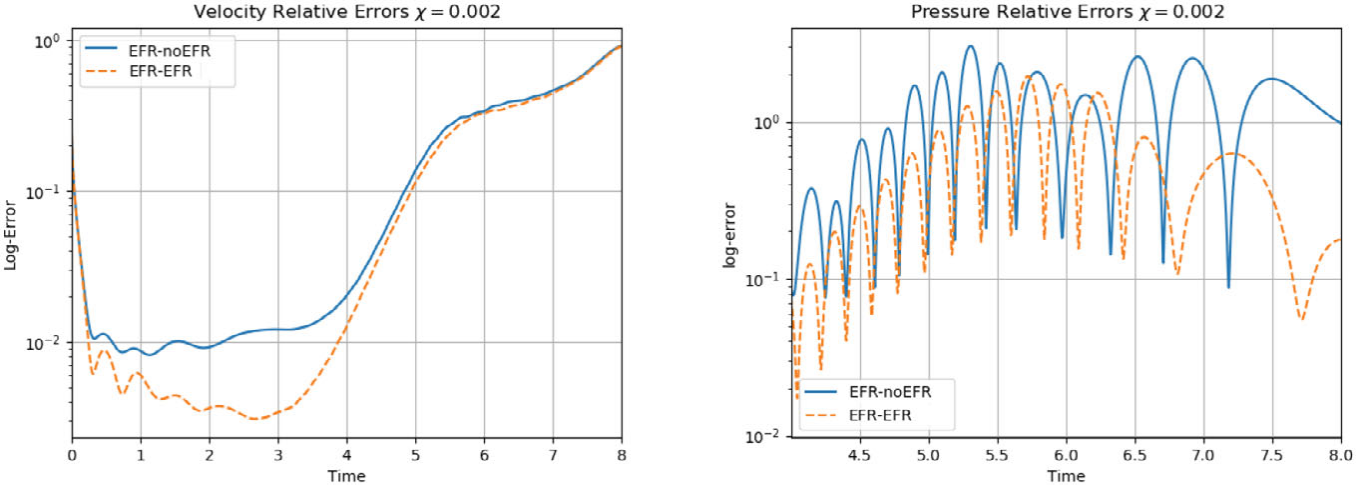
Experiment 3: δ=0.0032, χ=0.002 and $r_{u}=47$, $r_{p}=r_{s}=7$. *Left*: comparison of relative log‐errors over time for the velocity profiles: EFR‐noEFR (solid blue line) and EFR‐EFR (dashed orange line). *Right*: comparison of relative log‐errors over time for the pressure profiles: EFR‐noEFR (solid blue line) and EFR‐EFR (dashed orange line)

Overall, in Experiment 3, neither EFR‐noEFR nor EFR‐EFR give satisfactory results. Furthermore, both EFR‐noEFR and EFR‐EFR are significantly less accurate in Experiment 3 than in Experiment 2. Indeed, the log‐relative errors for the velocity field reported in Figure 13 (left) show that, even if the EFR‐EFR performs better on the first half of the time interval, both the EFR‐noEFR and the EFR‐EFR are inaccurate on the second half of the time interval.

**FIGURE 14:**
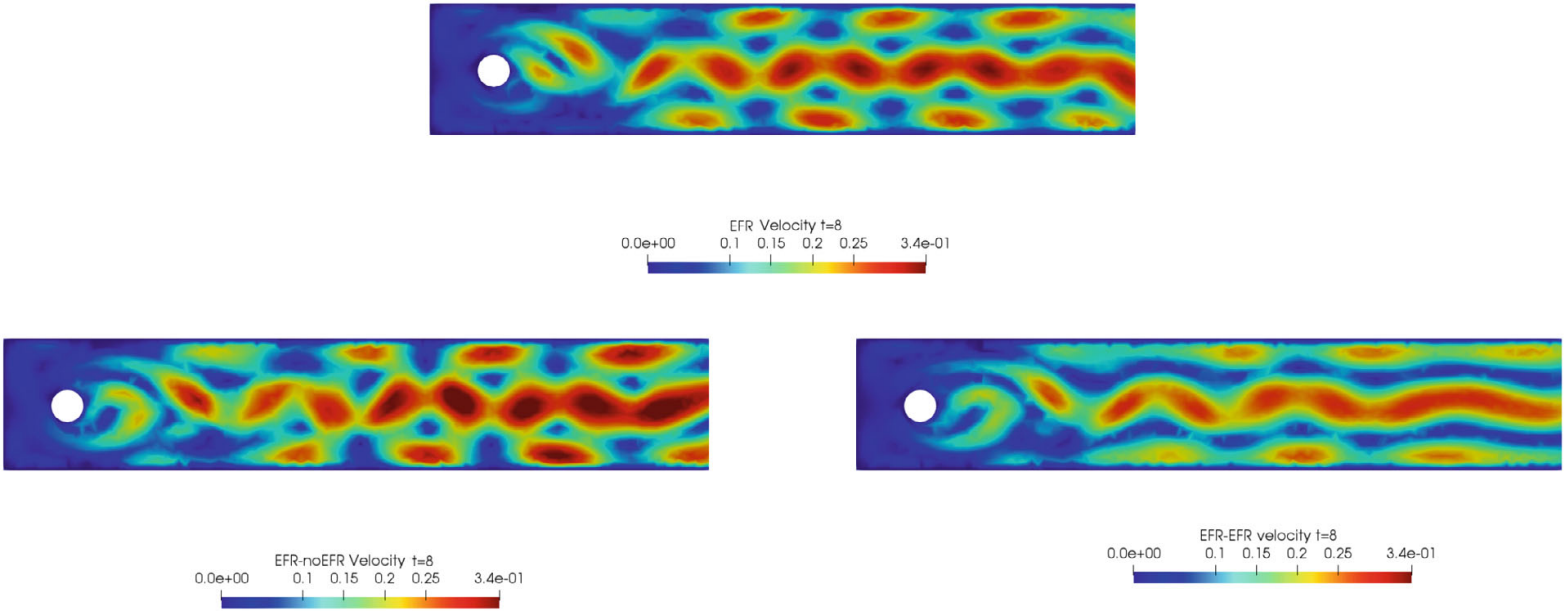
Experiment 3: δ=0.0032, χ=0.002, $r_{u}=47$, $r_{p}=r_{s}=7$ and t=8. *Top*: full order EFR velocity magnitude. *Bottom left*: reduced EFR‐noEFR velocity magnitude. *Bottom right*: reduced EFR‐EFR velocity magnitude

**FIGURE 15:**
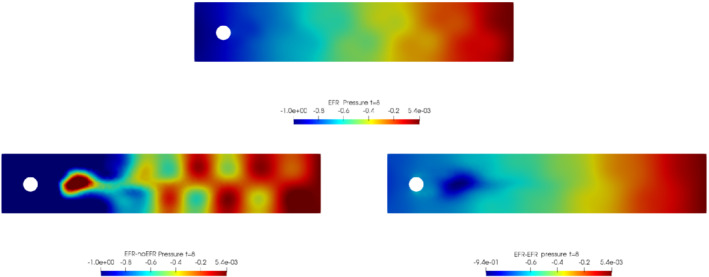
Experiment 3: δ=0.0032, χ=0.002, $r_{u}=47$, $r_{p}=r_{s}=7$ and t=8. *Top*: full order EFR pressure field. *Bottom left*: reduced EFR‐noEFR pressure field. *Bottom right*: reduced EFR‐EFR pressure field

**FIGURE 16:**
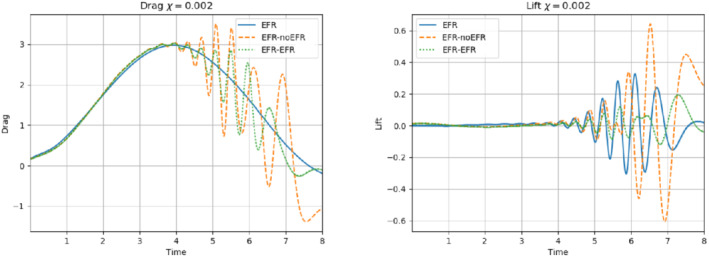
Experiment 3: δ=0.0032, χ=0.002 and $r_{u}=47$, $r_{p}=r_{s}=7$.) *Left*: $C_{D}(t)$ comparison over time: all the approaches almost coincide. *Right*: $C_{L}(t)$ comparison over time

This is confirmed by the velocity solution for t=8 displayed in Figure 14. The plot in Figure 13 (left) shows that, while for t∈[0,4] EFR‐EFR yields relative errors below $10^{-2}$ for the velocity field, this advantage is lost in the last part of the time interval, reaching unacceptable errors values (close to 1). On the other hand, concerning the pressure field (see Figure 13(right)), EFR‐EFR is able to perform better than EFR‐noEFR for almost the entire time window. However, the error values are high, even greater than 1 for some time instances. Moreover, the pressure field presents a checkerboard type of instability (although EFR‐noEFR model is inf‐sup stable thanks to supremizer stabilization) and the reconstruction is inaccurate, as displayed in Figure 15. These issues are visible also in Figure 16: the lift coefficient $C_{L}(t)$ is well recovered only for the first part of the time interval, when the vortex shedding does not occur and the low frequency modes are dominant. Even the drag coefficient $C_{D}(t)$ is not reconstructed in a satisfactory way and shows spurious oscillations for t>4. This behavior is worse in the EFR‐noEFR results, which exhibit larger amplitude oscillations. The EFR‐noEFR and EFR‐EFR yield the following $L^{2}-$errors of the force coefficients: ${\bar{E}}_{C_{L}}=2.39$, ${Ê}_{C_{L}}=1.2$, ${\bar{E}}_{C_{D}}=0.33$, and ${Ê}_{C_{D}}=0.15$. Overall, although the EFR‐EFR results are significantly more accurate than the EFR‐noEFR results, both ROMs yield relatively inaccurate results. These results show that, as expected, utilizing a more challenging regime (i.e., a coarser ROM resolution and mixed dynamics) in Experiment 3 deteriorates the EFR‐noEFR and EFR‐EFR performance. We emphasize, however, that even in this more challenging regime EFR‐EFR performs better than EFR‐noEFR.

### 3.5 Numerical results: Predictive regime

This section focuses on preliminary results on the predictive capabilities of the EFR‐noEFR and EFR‐EFR algorithms. We stress that the offline phase and the parameters χ and δ do not change with respect to the reconstructive setting although this choice may be suboptimal. In this section, we answer the following questions: (i) are the EFR‐noEFR and EFR‐EFR algorithms predictive? (ii) Which algorithm performs better in the predictive regime?

**FIGURE 17:**
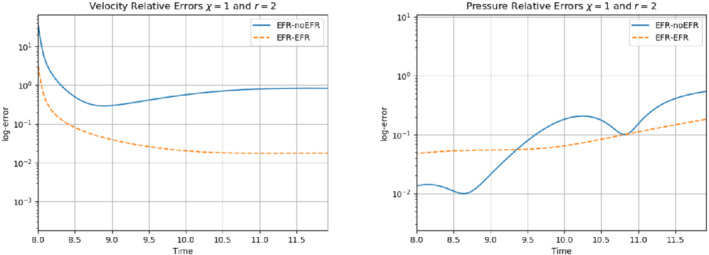
Experiment 1: δ=0.0032, χ=1 and r=2. Prediction for t∈[8,12]. *Left*: comparison of relative log‐errors over time of the velocity profiles: EFR full order versus EFR‐noEFR solutions and EFR full order versus EFR‐EFR solutions, represented by solid blue and dashed orange lines, respectively. *Right*: analogous representation for the relative log‐errors over time of the pressure profiles

**FIGURE 18:**
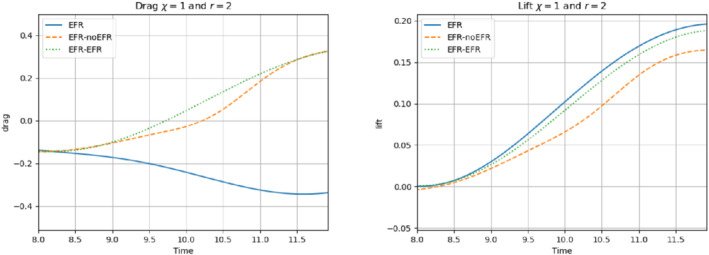
Experiment 1: δ=0.0032, χ=1 and r=2. Prediction for t∈[8,12]. *Left*: $C_{D}(t)$ comparison over time. *Right*: $C_{L}(t)$ comparison over time

**Experiment 1**. To study the predictability of ERF‐EFR and EFR‐noEFR strategies, we collect $N_{u}=N_{p}=200$ equally spaced snapshots for both velocity and pressure in [0,8]. After the POD procedure, we retain the first 2 modes for both velocity and pressure since they represent 99.9% of the snapshot energy. We recall that the filter radius is δ=0.0032 and the relaxation parameter is χ=1. We test the predictive capability of the model in the time interval [8,12]. In terms of relative velocity errors, EFR‐EFR performs better than EFR‐noROM, reaching values around $10^{-2}$ and reducing the error by two order of magnitude, as illustrated in the left plot of Figure 17. Focusing on the pressure field relative error, that is, the right plot of Figure 17, the EFR‐noEFR strategy performs better until t=9.4. After that value, the EFR‐noEFR error increases, while the EFR‐EFR remains stable around $10^{-1}$. For the sake of completeness, we report the $L^{2}$‐error values over the force coefficients: ${\bar{E}}_{C_{L}}=0.23$, ${Ê}_{C_{L}}=0.08$ and ${\bar{E}}_{C_{D}}=1.41$, ${Ê}_{C_{D}}=1.49$. These values are consistent with Figure 18, where the EFR‐EFR lift representation is more accurate than the EFR‐noEFR one, while the opposite happens for the drag coefficient. Table 4 lists maximum, minimum, and average error values over time for the velocity and pressure fields. This table confirms that, overall, EFR‐EFR is more accurate than EFR‐noEFR. The only exception is the minimum error for the pressure field, which is smaller for the EFR‐noEFR strategy, as already pointed out in analyzing Figure 17. The numerical results for Experiment 1 yield the following conclusions: (i) both approaches are predictive in time, and (ii) EFR‐EFR is, overall, more accurate than EFR‐noEFR, except for the drag representation.

**TABLE 4:** Experiment 1: δ=0.0032, χ=1, r=2

	EFR‐noEFR			EFR‐EFR		
	Maximum	Minimum	Average	Maximum	Minimum	Average
$E_{u}(t)$	1.239e+1	2.891e‐1	8.466e‐1	1.157e+0	1.739e‐2	5.692e‐2
$E_{p}(t)$	5.508e‐1	9.898e‐3	1.613e‐1	1.818e‐1	4.814e‐2	8.480e‐2

*Note*: Prediction for t∈[8,12]. Maximum, minimum, and average relative error over the considered time interval for velocity and pressure fields.

**Experiment 2**. The next step is represented by the analysis of the predictive regime in the setting of Experiment 2. Namely, we collect $N_{u}=N_{p}=2000$ equally spaced snapshots in the time interval [4,8]. We employ $r_{u}=43$, $r_{s}=r_{p}=8$ to retain 99.9% of the snapshots energy. In this case δ=0.0032 and χ=0.002.

**FIGURE 19:**
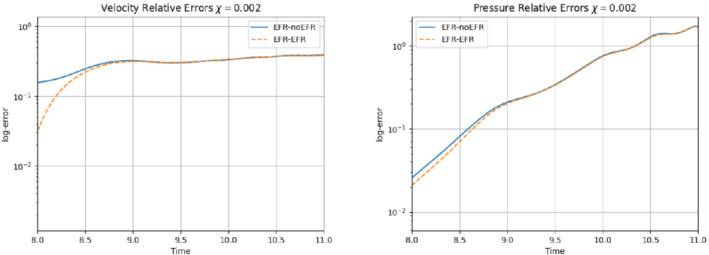
Experiment 2: δ=0.0032, χ=0.002 and $r_{u}=43,r_{p}=r_{s}=8$. Prediction for t∈[8,11]. *Left*: comparison of relative log‐errors over time of the velocity profiles: EFR full order versus EFR‐noEFR solutions and EFR full order versus EFR‐EFR solutions, represented by solid blue and dashed orange lines, respectively. *Right*: analogous representation for the relative log‐errors over time of the pressure profiles

**FIGURE 20:**
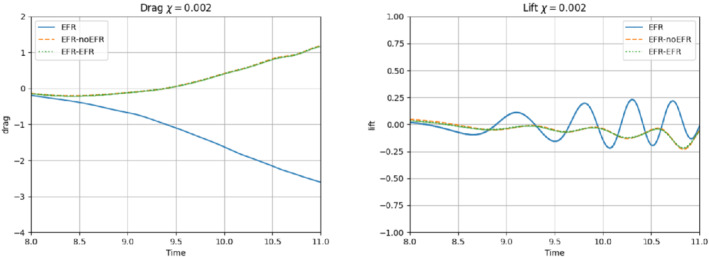
Experiment 2: δ=0.0032, χ=0.002 and $r_{u}=43,r_{p}=r_{s}=8$. Prediction for t∈[8,11].) *Left*: $C_{D}(t)$ comparison over time. *Right*: $C_{L}(t)$ comparison over time

We analyze the predictive regime up to T=11. We do not go further in time, since for t>11 the Newton's solver of the FOM simulation does not converge. From the plots in Figures 19 and 20, it is clear that EFR‐noEFR and EFR‐EFR are comparable. In the relative error plots of Figure 19, we see how both approaches struggle to represent velocity and pressure fields for large time values. Moreover, EFR‐noEFR and EFR‐EFR are not capable to accurately predict the force coefficients, as illustrated in Figure 20. These results are, respectively, confirmed by Table 5 and by the $L^{2}-$error over the force coefficients: ${\bar{E}}_{C_{L}}=1.00$, ${Ê}_{C_{L}}=1.00$, and ${\bar{E}}_{C_{D}}=0.99$, ${Ê}_{C_{D}}=0.99$. EFR‐EFR performs slightly better than EFR‐noEFR for all criteria, except for the drag coefficient. We note that, as in Experiment 3, both the EFR‐EFR and the EFR‐noEFR approaches struggle since the flow we investigate displays mixed dynamics (more complex dynamics in the time interval [4,8] and more laminar dynamics in [8, 11]).

**TABLE 5:** Experiment 2: δ=0.0032, χ=0.002, $r_{u}=43,r_{p}=r_{s}=8$

	EFR‐noEFR			EFR‐EFR		
	Maximum	Minimum	Average	Maximum	Minimum	Average
$E_{u}(t)$	1.000e+0	1.558e‐1	4.549e‐1	1.000e+0	3.211e‐2	4.437e‐1
$E_{p}(t)$	1.721e+0	2.569e‐2	6.856e‐1	1.704e+0	2.040e‐2	6.779e‐1

*Note*: Prediction for t∈[8,11]. Maximum, minimum, and average relative error over the considered time interval for velocity and pressure fields.

Overall, we conclude that (i) both the EFR‐EFR and the EFR‐noEFR approaches struggle in the predictive regime, and (ii) both approaches are comparable in terms of accuracy with respect to all the criteria. Finally, we also note that we do not investigate the EFR‐EFR and EFR‐noEFR algorithms in the predictive regime of the more challenging Experiment 3 since the two approaches struggled in the predictive regime of Experiment 2.

## 4 MODEL REDUCTION: WITH RESPECT TO TIME AND THE REYNOLDS NUMBER

In Section 3, we showed that the EFR‐EFR is more accurate than the EFR‐noEFR when model reduction is performed in the time domain. In this section, we perform a numerical investigation of the EFR‐EFR and EFR‐noEFR when model reduction is performed not only in the time domain (as we did in Section 3), but also in the parameter domain (i.e., with respect to ν). To this end, we consider the NSE (1) with a variable kinematic viscosity: $\nu \in [\nu_{\min},\nu_{\max}]\subset {\mathbb{R}}^{+}$. From definition (2), it is clear that changing ν will change the Reynolds number, which will vary in the interval $[0,Re_{\max}]\subset {\mathbb{R}}^{+}$.

To perform the model reduction both in the time domain and in the parameter domain, a standard POD approach that performs a simultaneous compression in time and in the parametric space would require a significant computational effort. Thus, to avoid the high computational cost of this brute force POD approach, in our numerical investigation we use a nested‐POD (n‐POD) algorithm. This compression algorithm is very popular (and goes by different names) in the ROM community: see, for example, References 71, 72, 73, 74. In the n‐POD algorithm, the compression is performed in two different stages, which operate first in time and then in the parametric space. Namely, first each time trajectory related to the parameter set explored is compressed, and then a POD reduction is performed on the already reduced parametric solutions. By decoupling the model reduction in time from the model reduction in the parameter domain, the n‐POD algorithm achieves significant reductions in computational time and storage with respect to the monolithic POD algorithm. In our numerical investigation, we use the n‐POD algorithm presented in Reference 75. The n‐POD is based on two decoupled levels.

1. *A first compression of the time trajectories*. In this first phase, a training set over the parameter space is chosen: ${\left\{\nu_{i}\right\}}_{i=1}^{N_{\nu }}$. For each $\nu_{i}$, a standard POD in time is applied, retaining the first $N_{u}^{t},N_{p}^{t},N_{s}^{t}$ modes for the velocity, pressure, and supremizer variables, respectively. We denote these modes scaled by their singular values with $m_{u(\nu_{i})}^{j},m_{p(\nu_{i})}^{j}$ and $m_{S(p(\nu_{i}))}^{j}$, for $j=1,\ldots ,N^{t}≐N_{u}^{t}=N_{p}^{t}=N_{s}^{t}$. For the sake of simplicity, in our setting we choose the same number of modes for all the variables and for all the parameters in the training set. However, in principle, one can choose the number of modes for each parametric snapshot through energy criteria, and the number can be different for each variable.
2. *A global compression of the scaled modes*. Using the POD procedure presented in Section 3, the following reduced space for velocity
$${𝕌}^{r_{us}}≐\text{POD}({\left\{m_{u(\nu_{1})}^{j}\right\}}_{j=1}^{N^{t}},\ldots ,{\left\{m_{u(\nu_{N_{\nu }})}^{j}\right\}}_{j=1}^{N^{t}})⊕\text{POD}({\left\{m_{S(p(\nu_{1}))}^{j}\right\}}_{j=1}^{N^{t}},\ldots ,{\left\{m_{S(p(\nu_{N_{\nu }}))}^{j}\right\}}_{j=1}^{N^{t}}),$$
  and the following space for pressure
$${\mathbb{Q}}^{r_{p}}≐\text{POD}({\left\{m_{p(\nu_{1})}^{j}\right\}}_{j=1}^{N^{t}},\ldots ,{\left\{m_{p(\nu_{N_{\nu }})}^{j}\right\}}_{j=1}^{N^{t}})$$
  are obtained.

For the sake of readability, we introduce the following notation

$${𝕌}^{r}≐\text{n‐POD}({\left\{u(\nu_{i})\right\}}_{i=1}^{N_{\nu }};N^{t})⊕\text{n‐POD}({\left\{S(p(\nu_{i}))\right\}}_{i=1}^{N_{\nu }};N^{t})$$

and

$${\mathbb{Q}}^{r_{p}}≐\text{n‐POD}({\left\{p(\nu_{i})\right\}}_{i=1}^{N_{\nu }};N^{t})$$

for the ROM velocity and pressure fields, respectively. Here, $N^{t}$ denotes the first phase of the time evolution compression, while *r* and $r_{p}$ are the final reduced space dimensions for velocity and pressure, after supremizer stabilization.

Pseudocodes that describe the EFR‐noEFR and EFR‐EFR approaches coupled with the n‐POD algorithm are reported in Algorithms 3 and 4, respectively.

Algorithm 3 **Pseudocode for EFR‐noEFR with n‐POD**

1

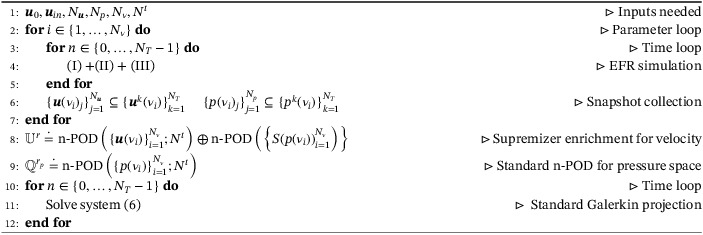

Algorithm 4 **Pseudocode for EFR‐EFR with n‐POD**

1

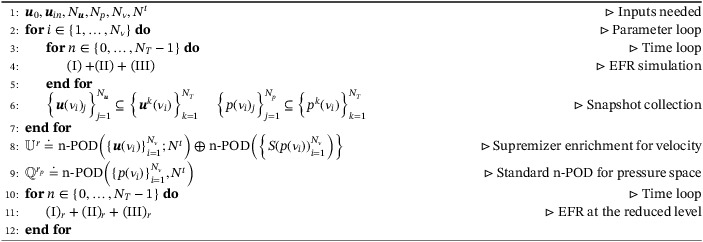

**Experiment 4**. In this experiment, we use the same test problem as the one used in Section 3.4 generalized to a parametric kinematic viscosity.

In our numerical investigation, we consider the parametric domain $\nu \in [10^{-3},1.575\times 10^{-3}]$, which yields $Re_{\max}\in [65,100]$. To explain the rationale for choosing this parametric domain, we define the averaged Reynolds number as

(21) $$\bar{Re}≐\frac{\bar{U}L}{\nu },$$

where $\bar{U}$ is the time averaged magnitude of the inflow velocity, $u_{in}$. For our parametric domain, we choose $\nu =1.575\times 10^{-3}$ since this value yields , which is the lower bound of the kinematic viscosity that achieves a vortex shedding behavior in the case of a steady inlet condition (see, e.g., Reference 76). We note that we choose a parametric domain that ensures only one type of flow dynamics (i.e., a vortex shedding regime). Choosing a parametric domain that spans various flow dynamics would be a more challenging test for the proposed ROMs, as noted in Experiment 3.

In the numerical investigation in Section 3, we used the Kolmogorov scale^62^, ^63^ as a filtering radius. However, the Kolmogorov scale changes with respect to the choice of the kinematic viscosity, ν. Thus, in Experiment 4, we use $\delta =h_{\min}$, that is, a classical choice for nonuniform meshes, as specified in Remark 4. We also fix χ=0.002. To build the ROM basis, we collect $N_{u}=N_{p}=2000$ equally spaced snapshots in the time interval [4,8], which is the setting used in Experiment 2. In the n‐POD algorithm, we perform the first compression choosing $N^{t}=60$ for each sampled parametric instance. We pick $N_{\nu }=20$ parameters using a log‐equispaced distribution. We choose the log‐uniform distribution since it allows us to collect more snapshots around the value $\nu =10^{-3}$, where we believe more information is needed because of higher vortex shedding frequency. We choose the $N^{t}$ value heuristically, seeking an accurate approximation of the time evolution for the various parametric snapshots. We pick $N_{\nu }=20$ seeking to minimize the computational costs of the building phase. Of course, a more detailed investigation would probably find better parameters, that is, parameters that yield more accurate approximations. In the second stage of compression of the n‐POD algorithm, we retain 99.9% of the system energy employing $r_{u}=33$, $r_{s}=r_{p}=3$.

In Section 4.1, we compare the EFR‐noEFR with the EFR‐EFR for $Re_{\max}=65$ (i.e., $\nu =1.575\times 10^{-3}$). In Section 4.2, we compare the EFR‐noEFR with the EFR‐EFR for $Re_{\max}=100$ (i.e., $\nu =10^{-3}$).

### 4.1 Reynolds number $Re_{\max}=65$: Reconstructive regime

**FIGURE 21:**
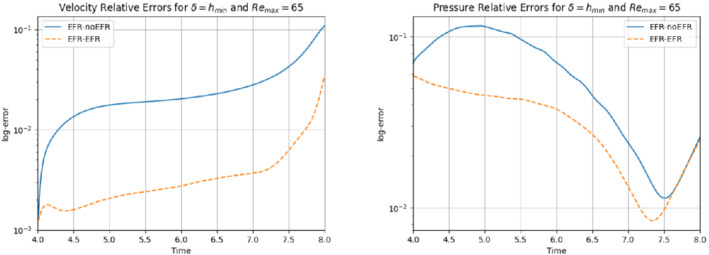
Experiment 4: $\delta =h_{\min}$, χ=0.002 and $r_{u}=33,r_{p}=r_{s}=3$, $Re_{\max}=65$. Reconstruction for t∈[4,8].) *Left*: comparison of relative log‐errors of the velocity profiles: EFR‐noEFR (solid blue line) and EFR‐EFR (dashed orange line). *Right*: comparison of relative log‐errors of the pressure profiles: EFR‐noEFR (solid blue line) and EFR‐EFR (dashed orange line)

**FIGURE 22:**
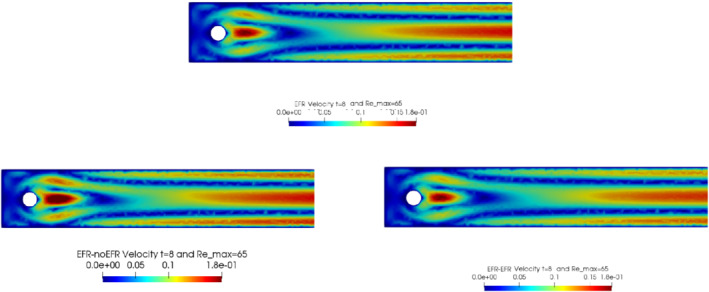
Experiment 4: $\delta =h_{\min}$, χ=0.002 and $r_{u}=33,r_{p}=r_{s}=3$, $Re_{\max}=65$, and t=8. *Top*: full order EFR velocity magnitude. *Bottom left*: reduced EFR‐noEFR velocity magnitude. *Bottom right*: reduced EFR‐EFR velocity magnitude

**FIGURE 23:**
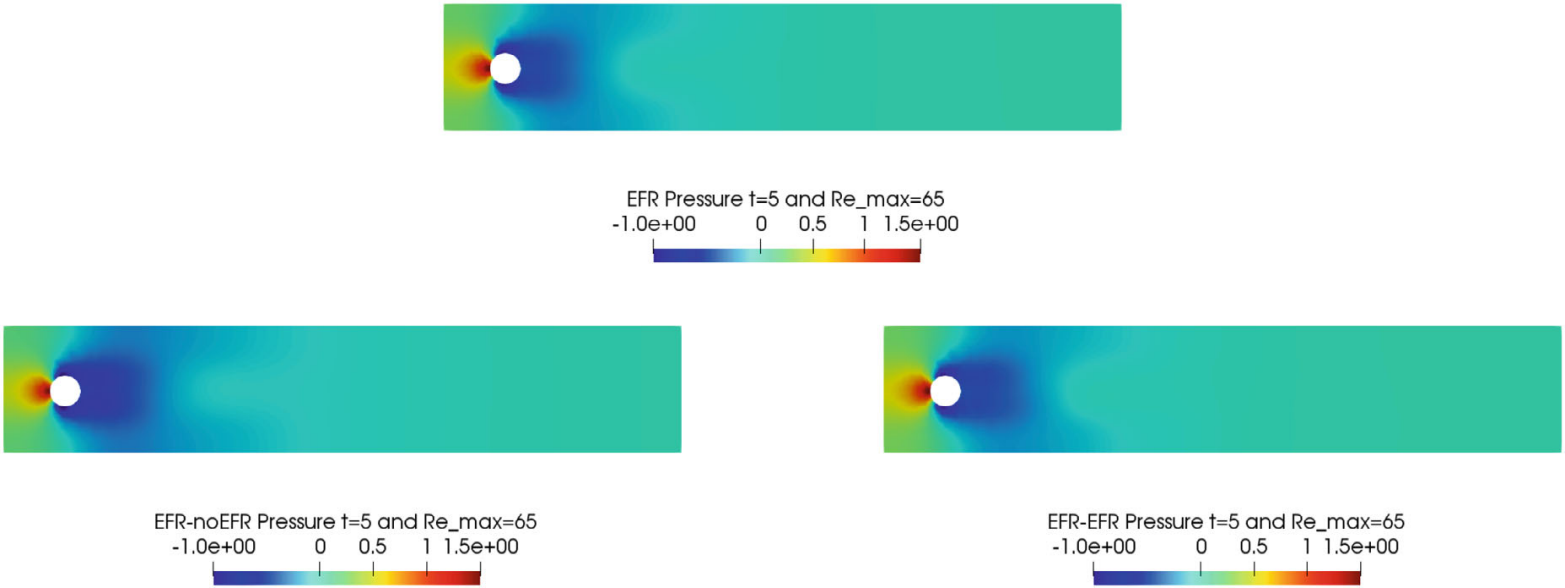
Experiment 4: $\delta =h_{\min}$, χ=0.002 and $r_{u}=33,r_{p}=r_{s}=3$, $Re_{\max}=65$, and t=5. *Top*: full order EFR pressure field. *Bottom left*: reduced EFR‐noEFR pressure field. *Bottom right*: reduced EFR‐EFR pressure field

**FIGURE 24:**
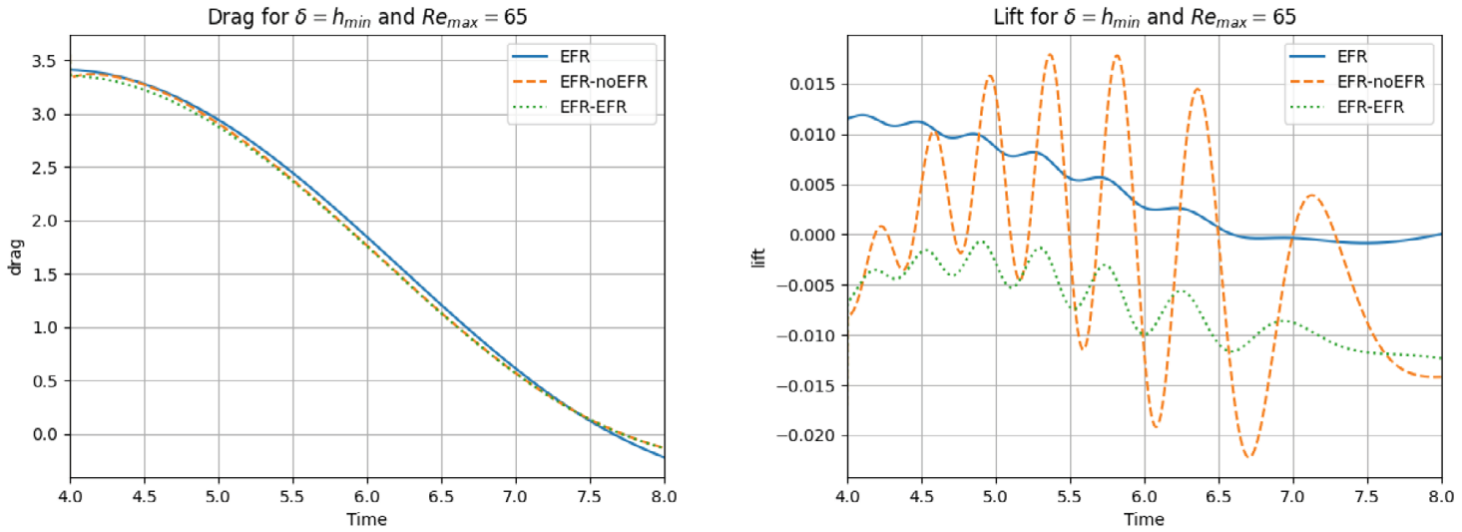
Experiment 4: $\delta =h_{\min}$, χ=0.002 and $r_{u}=33,r_{p}=r_{s}=3$, $Re_{\max}=65$. Reconstruction for t∈[4,8].) *Left*: $C_{D}(t)$ comparison over time. *Right*: $C_{L}(t)$ comparison over time

The relative log‐errors plotted in Figure 21 for both the velocity and the pressure are lower for the EFR‐EFR than for the EFR‐noEFR. The EFR‐EFR is also more accurate than the EFR‐noEFR in approximating the velocity field at t=8 (Figure 22) and the pressure field at t=5 (Figure 23). Both algorithms yield accurate approximations for the drag coefficient, $C_{D}(t)$ (Figure 24, left) and relatively inaccurate approximations for the lift coefficient, $C_{L}(t)$ (Figure 24, right). Table 6 lists the maximum, minimum, and average error values over time for the velocity and pressure fields, and shows that the EFR‐EFR is more accurate than the EFR‐noEFR. However, the EFR‐noEFR strategy is slightly more accurate in the reconstruction of the force coefficients in terms of $L^{2}-$errors: ${\bar{E}}_{C_{L}}=1.75$, ${Ê}_{C_{L}}=1.84$, ${\bar{E}}_{C_{D}}=0.025$, and ${Ê}_{C_{D}}=0.030$.

**TABLE 6:** Experiment 4: $\delta =h_{\min}$, χ=0.002, $r_{u}=33,r_{p}=r_{s}=3$, and $Re_{\max}=65$

	EFR‐noEFR			EFR‐EFR		
	Maximum	Minimum	Average	Maximum	Minimum	Average
$E_{u}(t)$	1.086e‐1	1.229e‐3	2.636e‐1	3.635e‐2	1.230e‐3	4.269e‐3
$E_{p}(t)$	1.157e‐1	1.136e‐2	6.507e‐1	6.316e‐2	8.833e‐3	3.651e‐2

*Note*: Reconstruction for t∈[4,8]. Maximum, minimum, and average relative error over the considered time interval for the velocity and pressure fields.

**FIGURE 25:**
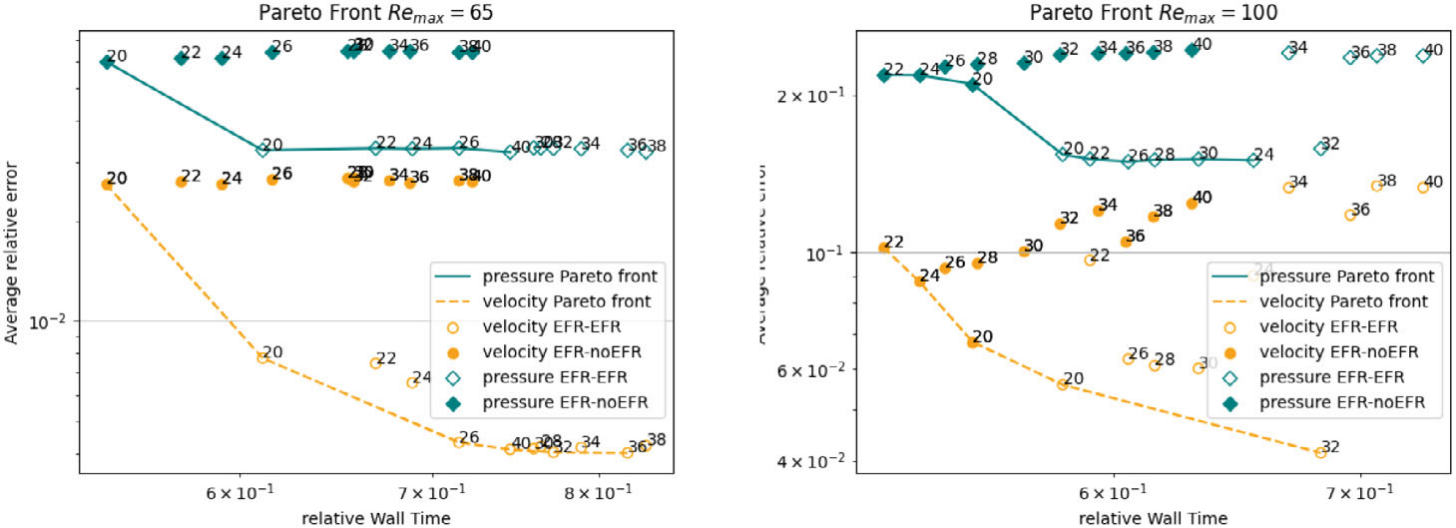
Experiment 4: $\delta =h_{\min}$, χ=0.002, $r_{u}=\{20,22,24,26,28,30,32,34,36,38,40\}$, and $r_{p}=r_{s}=3$. *Left*: pareto plots for velocity (orange) and pressure (teal) fields and $Re_{\max}=65$: averaged relative error in time versus relative wall time for varying $r_{u}$. *Right*: analogous plot for $Re_{\max}=100$

Overall, the improvement in the EFR‐EFR is highlighted by the left Pareto plot in Figure 25. Indeed, fixing $r_{p}=r_{s}=3$ and choosing $r_{u}=20,22,24,26,28,30,32,34,36,38,40$ shows that, over this range of $r_{u}$ values, the EFR‐EFR performs better than the EFR‐noEFR with respect to both the velocity and the pressure approximations. Indeed, the EFR‐EFR with $r_{u}=20$ yields a low relative error that the EFR‐noEFR cannot attain by increasing its $r_{u}$ value (and, consequently, its relative wall time).

### 4.2 Reynolds number $Re_{\max}=100$: Reconstructive regime

**FIGURE 26:**
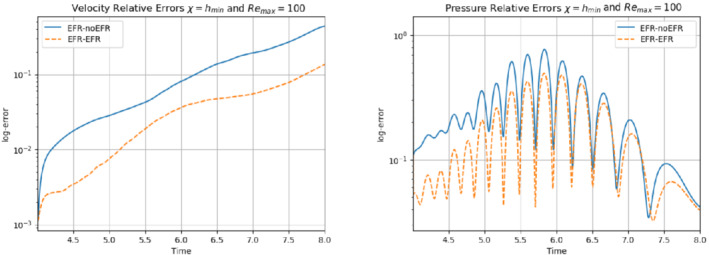
Experiment 4: $\delta =h_{\min}$, χ=0.002, $r_{u}=33,r_{p}=r_{s}=3$, and $Re_{\max}=100$. Reconstruction for t∈[4,8]. *Left*: comparison of relative log‐errors of the velocity profiles: EFR‐noEFR (solid blue line) and EFR‐EFR (dashed orange line). *Right*: comparison of relative log‐errors of the pressure profiles: EFR‐noEFR (solid blue line) and EFR‐EFR (dashed orange line)

**FIGURE 27:**
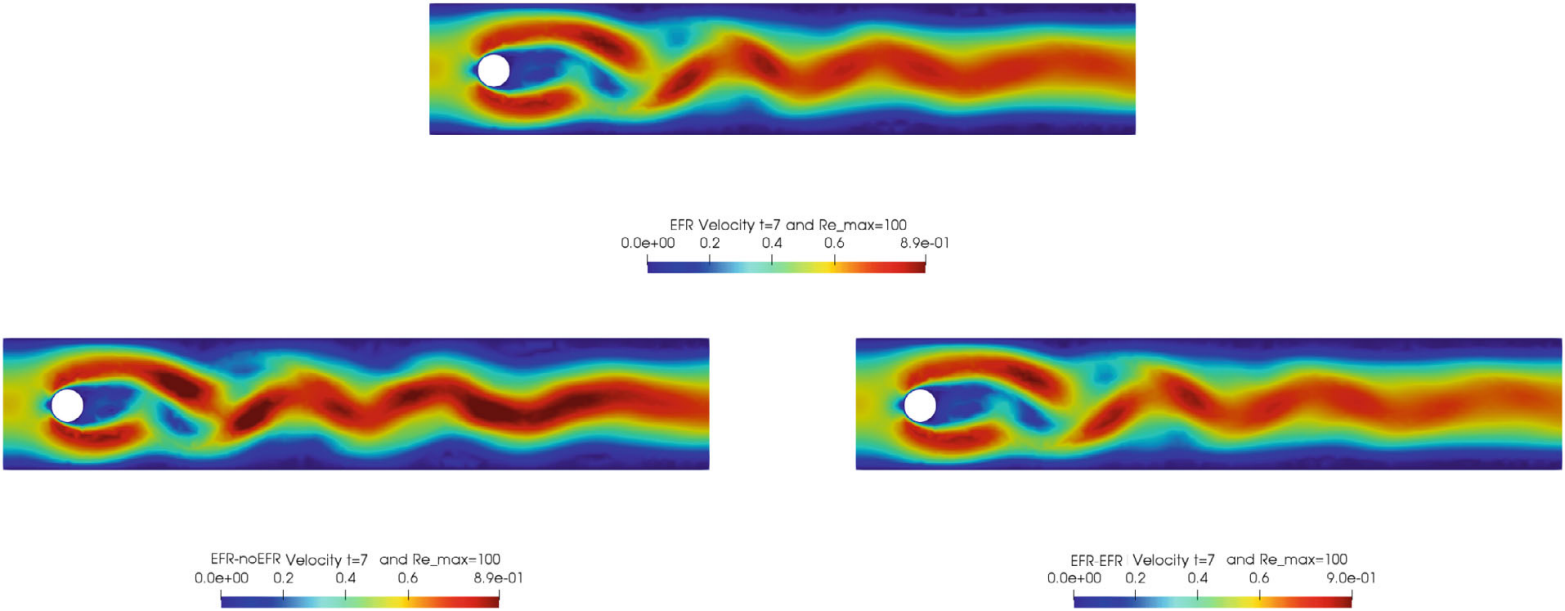
Experiment 4: $\delta =h_{\min}$, χ=0.002 and $r_{u}=33,r_{p}=r_{s}=3$, $Re_{\max}=100$, and t=7. *Top*: full order EFR velocity magnitude. *Bottom left*: reduced EFR‐noEFR velocity magnitude. *Bottom right*: reduced EFR‐EFR velocity magnitude

**FIGURE 28:**
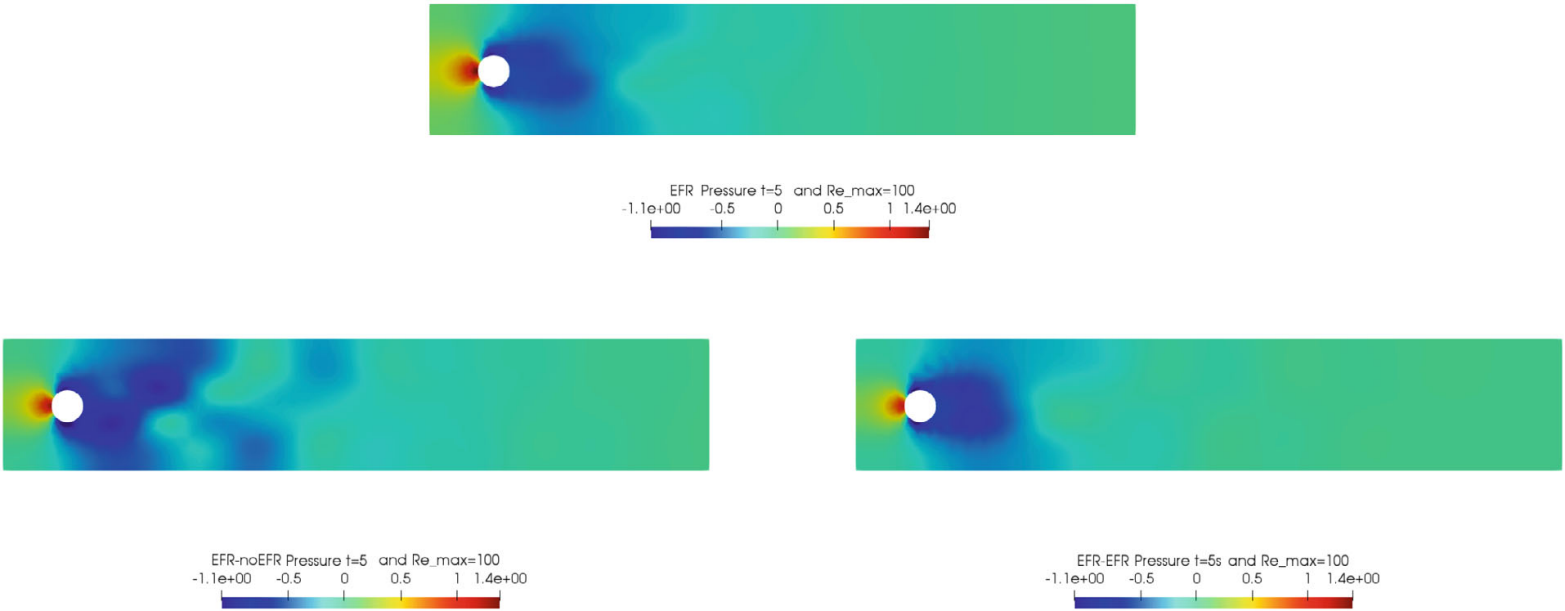
Experiment 4: $\delta =h_{\min}$, χ=0.002 and $r_{u}=33,r_{p}=r_{s}=3$, $Re_{\max}=100$, and t=5. *Top*: full order EFR pressure field. *Bottom left*: reduced EFR‐noEFR pressure field. *Bottom right*: reduced EFR‐EFR pressure field

**FIGURE 29:**
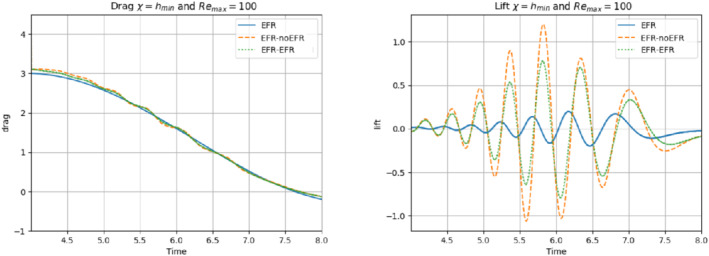
Experiment 4: $\delta =h_{\min}$, χ=0.002, $r_{u}=33,r_{p}=r_{s}=3$, and $Re_{\max}=100$. Reconstruction for t∈[4,8]. *Left*: $C_{D}(t)$ comparison over time. *Right*: $C_{L}(t)$ comparison over time

The relative log‐errors plotted in Figure 26 for both the velocity and the pressure are significantly lower for the EFR‐EFR than for the EFR‐noEFR. The EFR‐EFR is also more accurate than the EFR‐noEFR in approximating the velocity field at t=7 (Figure 27) and the pressure field at t=5 (Figure 28). Furthermore, both the EFR‐EFR and the EFR‐noEFR yield accurate drag coefficients, $C_{D}(t)$ (Figure 29, left). Although both the EFR‐EFR and the EFR‐noEFR lift coefficients, $C_{L}(t)$ (Figure 29, right), are relatively inaccurate, the EFR‐EFR approximation is more accurate than the EFR‐noEFR.

Focusing on Table 7, we observe that EFR‐EFR and EFR‐noEFR present comparable results for the minimum value of the velocity relative error. For the other pointwise error values, EFR‐EFR is more accurate than EFR‐noEFR, both for velocity and pressure. In terms of $L^{2}-$errors of the force coefficients, EFR‐EFR is always more accurate than EFR‐noEFR. Indeed, ${\bar{E}}_{C_{L}}=5.55$, ${Ê}_{C_{L}}=4.08$, ${\bar{E}}_{C_{D}}=0.04$, and ${Ê}_{C_{D}}=0.02$. We also present a Pareto plot for $Re_{\max}=100$ in the right panel of Figure 25. We fix $r_{p}=r_{s}=3$ and choose $r_{u}=20,22,24,26,28,30,32,34,36,38,40$. Over this range of $r_{u}$ values, we observe that, although EFR‐noEFR is optimal for smaller $r_{u}$ values, the relatively low EFR‐EFR error for $r_{u}=20$ cannot be reached by EFR‐noEFR even by increasing its $r_{u}$ value (and, consequently, its relative wall time).

**TABLE 7:** Experiment 4: $\delta =h_{\min}$, χ=0.002, $r_{u}=33,r_{p}=r_{s}=3$, and $Re_{\max}=100$

	EFR‐noEFR			EFR‐EFR		
	Maximum	Minimum	Average	Maximum	Minimum	Average
$E_{u}(t)$	4.383e‐1	1.115e‐3	1.220e‐1	1.354e‐1	1.117e‐3	3.871e‐2
$E_{p}(t)$	7.720e‐1	3.419e‐2	2.426e‐1	4.949e‐1	3.215e‐2	1.581e‐1

*Note*: Reconstruction for t∈[4,8]. Maximum, minimum, and average relative error over the considered time interval for velocity and pressure fields.

The numerical results for $Re_{\max}=65$ and $Re_{\max}=100$ show that, for both Reynolds numbers and for all criteria, the EFR‐EFR is consistently more accurate than the EFR‐noEFR (although this difference between the EFR‐EFR and EFR‐noEFR seems to be somewhat lower for $Re_{\max}=100$). Thus, the numerical investigation in this section suggests that the FOM‐ROM consistency is important when the EFR stabilization is used and model reduction is performed both in time and in the parametric domain.

### 4.3 Reynolds number $Re_{\max}=110$: Predictive regime

In this section, we analyze the predictive capabilities of the EFR‐EFR and EFR‐noEFR algorithms with respect to the Reynolds number. We note that we do not investigate the predictive capabilities of the two algorithms with respect to both time and Reynolds number since both algorithms struggled in the predictive regime for Experiment 2 in Section 3.5. We follow the approach used in Section 3.5 and try to answer the following questions: (i) are the EFR‐noEFR and EFR‐EFR algorithms predictive? (ii) Which algorithm performs better in the predictive regime with respect to the Reynolds number?

**FIGURE 30:**
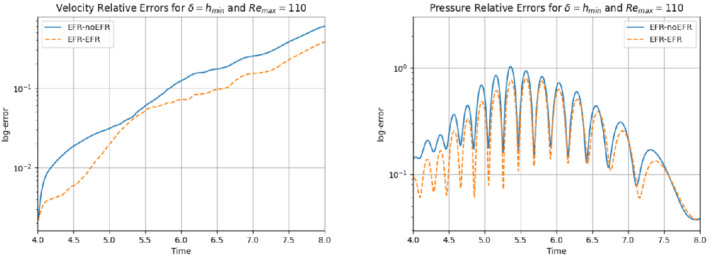
Experiment 4: $\delta =h_{\min}$, χ=0.002, $r_{u}=33$, and $r_{p}=r_{s}=3$. Prediction for $Re_{\max}=110$ in [4,8]. *Left*: comparison of relative log‐errors over time of the velocity profiles: EFR full order versus EFR‐noEFR solutions and EFR full order versus EFR‐EFR solutions, represented by solid blue and dashed orange lines, respectively. *Right*: Analogous representation for the relative log‐errors over time of the pressure profiles

We first present results for $Re_{\max}=110$. In our numerical investigation, we use the same computational setting as that used in Experiment 4. Specifically, we use $\delta =h_{\min}$ and χ=0.002. We also collect $N_{u}=N_{p}=2000$ equally spaced snapshots in the time interval [4,8] for $N_{\nu }=20$. For these snapshots, we choose a log‐equispaced distribution in the parametric domain $\nu \in [10^{-3},1.575\times 10^{-3}]$. We employ $r_{u}=33$ and $r_{s}=r_{p}=3$ to retain 99.9% of the snapshot energy. The relative error plots in Figure 30 show that EFR‐EFR is consistently more accurate than the EFR‐noEFR in approximating both the velocity and the pressure. We note, however, that both the EFR‐EFR and the EFR‐noEFR are relatively inaccurate in approximating the pressure field.

**FIGURE 31:**
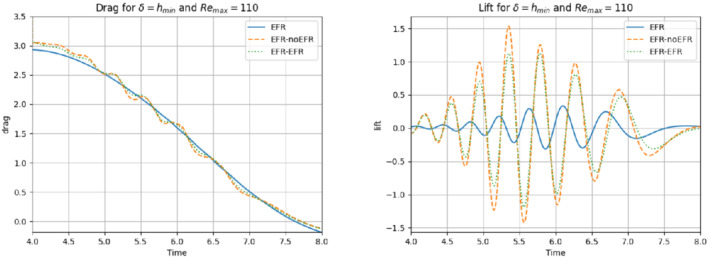
Experiment 4: $\delta =h_{\min}$, χ=0.002, $r_{u}=33$, and $r_{p}=r_{s}=3$. Prediction for $Re_{\max}=110$ in [4,8]. *Left*: $C_{D}(t)$ comparison over time. *Right*: $C_{L}(t)$ comparison over time

A similar behavior is observed with respect to the force coefficients, which are displayed in Figure 31: EFR‐EFR is consistently more accurate than the EFR‐noEFR. We note that the EFR‐EFR improvement over the EFR‐noEFR is only marginal for the drag coefficient. Furthermore, both the EFR‐EFR and the EFR‐noEFR produce relatively inaccurate approximations of the lift coefficient. The qualitative behavior of the plots in Figures 30 and 31 is supported by the maximum, minimum, and average relative errors listed in Table 8, and by the $L^{2}-$error of the force coefficients: ${\bar{E}}_{C_{L}}=4.77$, ${Ê}_{C_{L}}=3.99$, and ${\bar{E}}_{C_{D}}=0.048$, ${Ê}_{C_{D}}=0.037$.

Overall, these results yield the following conclusions: (i) the EFR‐EFR and EFR‐noEFR algorithms are predictive in the approximation of the velocity field and the drag coefficient, but both struggle in the approximation of the pressure field and the lift coefficient. (ii) The EFR‐EFR algorithm is consistently more accurate than the EFR‐noEFR algorithm with respect to all criteria, especially in the approximation of the velocity field.

**TABLE 8:** Experiment 4: $\delta =h_{\min}$, χ=0.002, $r_{u}=33$, and $r_{p}=r_{s}=3$

	EFR‐noEFR			EFR‐EFR		
	Maximum	Minimum	Average	Maximum	Minimum	Average
$E_{u}(t)$	5.943e‐1	2.138e‐3	1.636e‐1	3.752e‐1	2.146e‐3	9.869e‐2
$E_{p}(t)$	2.451e+0	3.758e‐2	3.247e‐1	2.451e+0	3.633e‐2	2.558e‐1

*Note*: Prediction for $Re_{\max}=110$ in [4,8]. Maximum, minimum, and average relative error over the considered time interval for the velocity and pressure fields.

### 4.4 Reynolds number $Re_{\max}=140$: Predictive regime

**FIGURE 32:**
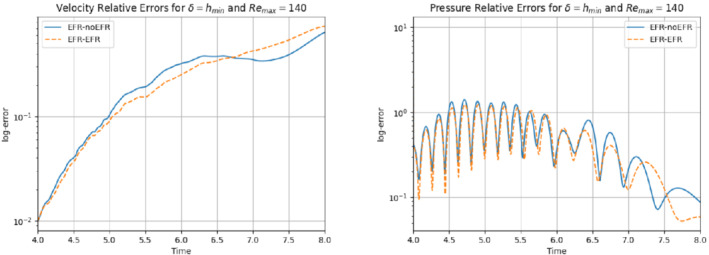
Experiment 4: $\delta =h_{\min}$, χ=0.002, $r_{u}=33$, and $r_{p}=r_{s}=3$. Prediction for $Re_{\max}=140$ in [4,8]. *Left*: Comparison of relative log‐errors over time of the velocity profiles: EFR full order versus EFR‐noEFR solutions and EFR full order versus EFR‐EFR solutions, represented by solid blue and dashed orange lines, respectively. *Right*: Analogous representation for the relative log‐errors over time of the pressure profiles

**FIGURE 33:**
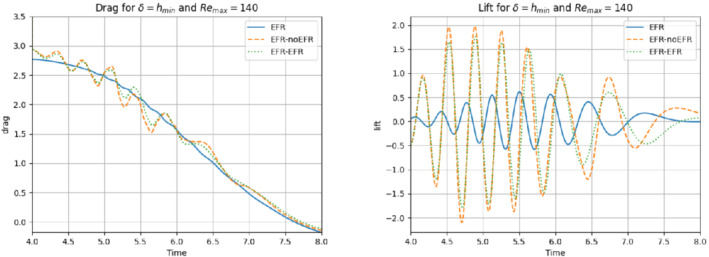
Experiment 4: $\delta =h_{\min}$, χ=0.002, $r_{u}=33$, and $r_{p}=r_{s}=3$. Prediction for $Re_{\max}=140$ in [4,8]. *Left*: $C_{D}(t)$ comparison over time. *Right*: $C_{L}(t)$ comparison over time

In this numerical investigation, we increase the Reynolds number to $Re_{\max}=140$ and use the same computational setting as that used in Experiment 4. Specifically, we use $\delta =h_{\min}$ and χ=0.002, and collect $N_{u}=N_{p}=2000$ equally spaced snapshots in the time interval [4,8] for $N_{\nu }=20$. For these snapshots, we choose a log‐equispaced distribution in the parametric domain $\nu \in [10^{-3},1.575\times 10^{-3}]$. We employ $r_{u}=33$ and $r_{s}=r_{p}=3$ to retain 99.9% of the snapshot energy. The relative error plots in Figure 32 show that the EFR‐EFR and EFR‐noEFR algorithms perform similarly: they predict accurately the velocity field at the beginning, but their accuracy starts to degrade toward the end of the time interval. Their predictions of the pressure field are inaccurate at the beginning of the simulation, but they become more accurate toward the end of the time interval. A similar behavior is observed with respect to the force coefficients, which are displayed in Figure 33. The EFR‐EFR and EFR‐noEFR algorithms perform similarly and provide relatively accurate approximations of the drag coefficient, but their approximations of the lift coefficient are inaccurate. The qualitative behavior of the plots in Figures 32 and 33 is supported by the maximum, minimum, and average relative errors listed in Table 9, and by the $L^{2}-$error of the force coefficients: ${\bar{E}}_{C_{L}}=3.91$, ${Ê}_{C_{L}}=3.64$, and ${\bar{E}}_{C_{D}}=0.098$, ${Ê}_{C_{D}}=0.11$. Overall, these results yield the following conclusions: (i) the EFR‐EFR and EFR‐noEFR algorithms are predictive in the approximation of the velocity field and the drag coefficient, but both struggle in the approximation of the pressure field and the lift coefficient. (ii) The EFR‐EFR and EFR‐noEFR algorithms perform similarly with respect to all criteria. We believe that, to increase the predictive capabilities of the EFR‐EFR and EFR‐noEFR algorithms, the parameters δ and χ should be tuned appropriately. This, however, goes beyond the scope of the current investigation.

**TABLE 9:** Experiment 4: $\delta =h_{\min}$, χ=0.002, $r_{u}=33$, and $r_{p}=r_{s}=3$

	EFR‐noEFR			EFR‐EFR		
	Maximum	Minimum	Average	Maximum	Minimum	Average
$E_{u}(t)$	6.387e‐1	9.922e‐3	2.617e‐1	7.310e‐1	9.930e‐3	2.768e‐1
$E_{p}(t)$	1.029e+1	7.233e‐2	5.289e‐1	1.029e+1	5.260e‐2	4.744e‐1

*Note*: Prediction for $Re_{\max}=140$ in [4,8]. Maximum, minimum, and average relative error over the considered time interval for velocity and pressure fields.

## 5 CONCLUSIONS

In this article, we took a step in the study of FOM‐ROM consistency when the EFR algorithm is used as numerical stabilization in convection‐dominated, marginally‐resolved flows. To this end, as a mathematical model we considered the incompressible Navier–Stokes equations. We used moderate Reynolds numbers, which yielded a convection‐dominated regime. We performed FOM and ROM simulations in the marginally‐resolved regime, that is, when the number of degrees of freedom is barely capable of capturing the main features of the underlying flow. To tackle the inaccuracies of the FOM and ROM simulations in the marginally‐resolved regime, we employed the EFR algorithm, which leverages spatial filtering to alleviate the spurious oscillations.

To investigate the FOM‐ROM consistency, we considered two models:

- the EFR‐noEFR, in which the EFR regularization is used at a FOM level, but not at a ROM level;
- the EFR‐EFR, in which the EFR regularization is used both at a FOM and at a ROM level.

We investigated the EFR‐noEFR and EFR‐EFR in the numerical simulation of a 2D flow past a circular cylinder at time‐dependent Reynolds numbers with a maximum value Re=100. As criteria for our comparison, we used the relative velocity error, the relative pressure error, and the lift and drag coefficients. We also considered two types of model reduction: (i) model reduction in time, for which we used the POD algorithm; and (ii) model reduction in time and in the parameter space, for which we used the nested‐POD algorithm. In all our tests, for both types of model reduction, and for all three criteria, the EFR‐EFR was more accurate than the EFR‐noEFR. These results suggest that FOM–ROM consistency is beneficial for the EFR regularization in a convection‐dominated, marginally‐resolved regime.

These first steps in the study of the FOM‐ROM consistency of regularized models are encouraging. There are, however, other research directions that should be investigated for a deeper understanding of this important topic. Probably the most important investigation should focus on the FOM‐ROM consistency for regularized models in the *under‐resolved* regime, which is important in many realistic settings (e.g., turbulent flows) where the ROM dimension is significantly lower than the number of degrees of freedom needed to accurately represent the complex dynamics of the underlying system. Related to this investigation, higher Reynolds number flows should be considered. Another important research direction is the investigation of FOM‐ROM consistency when *different* regularized models are used at the FOM and ROM levels (e.g., the Leray model is used at the FOM level and the EFR model is used at the ROM level). Related to this, one could also investigate the *parameter* FOM‐ROM consistency, which is complementary to the *model* FOM‐ROM consistency investigated in this article. Specifically, one could consider the same regularized model at the FOM and ROM levels, but use different parameters (e.g., different δ values) in these regularized models. Using different δ or χ values at the FOM and ROM levels (i.e., $\delta^{FOM}\neq \delta^{ROM}$ or $\chi^{FOM}\neq \chi^{ROM}$) could yield more accurate ROM solutions in several settings, such as hyper‐reduction.

Finally, we emphasize that most of the existing studies (including this article) on the FOM‐ROM consistency have been numerical investigations. Although theoretical investigations could support the existing numerical investigations and shed new light on the FOM‐ROM consistency, these studies are relatively scarce (for notable examples, see the numerical analysis performed in References 28 and 30 for FOM‐ROM consistency of the streamline upwind Petrov‐Galerkin (SUPG) stabilization, and Reference 51 for FOM‐ROM consistency with respect to the discretization of the nonlinearity of the Navier–Stokes equations). In a future study, we plan to perform the numerical analysis of the FOM‐ROM consistency with respect to the EFR regularization, and investigate whether the theoretical results support the numerical findings in the numerical investigation in this article.

These numerical and theoretical investigations of various types of FOM‐ROM consistency could provide a new impetus for the development of ROMs that are consistent with their corresponding FOMs.
